# MYC Inhibition Halts Metastatic Breast Cancer Progression by Blocking Growth, Invasion, and Seeding

**DOI:** 10.1158/2767-9764.CRC-21-0103

**Published:** 2022-02-21

**Authors:** Daniel Massó-Vallés, Marie-Eve Beaulieu, Toni Jauset, Fabio Giuntini, Mariano F. Zacarías-Fluck, Laia Foradada, Sandra Martínez-Martín, Erika Serrano, Génesis Martín-Fernández, Sílvia Casacuberta-Serra, Virginia Castillo Cano, Jastrinjan Kaur, Sergio López-Estévez, Miguel Ángel Morcillo, Mohammad Alzrigat, Loay Mahmoud, Antonio Luque-García, Marta Escorihuela, Marta Guzman, Joaquín Arribas, Violeta Serra, Lars-Gunnar Larsson, Jonathan R. Whitfield, Laura Soucek

**Affiliations:** 1 Preclinical & Translational Research Program, Vall d'Hebron Institute of Oncology (VHIO), Vall d'Hebron Barcelona Hospital Campus, C/ Natzaret, Barcelona, Spain.; 2 Peptomyc S.L., Vall d'Hebron Barcelona Hospital Campus, Barcelona, Spain.; 3 Department of Biochemistry and Molecular Biology, Universitat Autònoma de Barcelona, Bellaterra, Spain.; 4 Centro de Investigaciones Energéticas, Medioambientales y Tecnológicas (CIEMAT), Madrid, Spain.; 5 Department of Microbiology, Tumor and Cell Biology (MTC), Karolinska Institutet, Stockholm, Sweden.; 6 Institució Catalana de Recerca i Estudis Avançats (ICREA), Barcelona, Spain.

## Abstract

MYC's role in promoting tumorigenesis is beyond doubt, but its function in the metastatic process is still controversial. Omomyc is a MYC dominant negative that has shown potent antitumor activity in multiple cancer cell lines and mouse models, regardless of their tissue of origin or driver mutations, by impacting on several of the hallmarks of cancer. However, its therapeutic efficacy against metastasis has not been elucidated yet. Here we demonstrate for the first time that MYC inhibition by transgenic Omomyc is efficacious against all breast cancer molecular subtypes, including triple-negative breast cancer, where it displays potent antimetastatic properties both *in vitro* and *in vivo*. Importantly, pharmacologic treatment with the recombinantly produced Omomyc miniprotein, recently entering a clinical trial in solid tumors, recapitulates several key features of expression of the Omomyc transgene, confirming its clinical applicability to metastatic breast cancer, including advanced triple-negative breast cancer, a disease in urgent need of better therapeutic options.

**Significance:**

While MYC role in metastasis has been long controversial, this manuscript demonstrates that MYC inhibition by either transgenic expression or pharmacologic use of the recombinantly produced Omomyc miniprotein exerts antitumor and antimetastatic activity in breast cancer models *in vitro* and *in vivo*, suggesting its clinical applicability.

## Introduction

Breast cancer is the most common cancer in women and the second leading cause of cancer mortality, with more than 2.2 million annual cases and 685,000 estimated deaths globally (1). It is a highly heterogeneous disease divided into histologic and molecular subtypes (2). In clinical practice, patients are classified according to the expression of estrogen receptor (ER), progesterone receptor (PR), amplification of human epidermal growth factor receptor 2 (HER2), and proliferation rate (Ki67 positivity). These molecular subtypes differ in incidence, prognosis, and treatment options (3–5). Triple-negative breast cancers (TNBC) are a heterogeneous group of tumors, devoid of expression of ER, PR, and HER2, with one common feature: a distinctly aggressive nature with higher rates of relapse and shorter overall survival in the metastatic setting compared with other subtypes of breast cancer (6). Hence, their prognosis remains dire, with an average median survival of 1 year for metastatic disease (7).

Despite major improvements in prevention, diagnosis and treatment, many patients still develop metastatic breast cancer (mBC). Because of the selective pressure imposed on the primary tumors, metastases evolve and differentiate, sometimes acquiring characteristics of more aggressive subtypes (8). Therefore, most metastatic patients become resistant to therapy and eventually succumb to their disease. Regardless of the molecular subtype, mBC is essentially incurable, with only 1% to 3% of patients with chemotherapy-naïve mBC remaining disease-free over the long term after systemic therapy (9).

The *MYC* oncogene is amplified or deregulated in the majority of human cancers (10). In breast cancer, amplification and overexpression of *MYC* are frequent in high grade and invasive malignancies and are consistently correlated with poor outcome and early recurrence (11–14). Even distant lethal breast cancer metastases from *MYC*-unamplified primary tumors often acquire *MYC* amplification (15). Interestingly, MYC is disproportionately elevated in TNBC compared with ER/PR^+^ and HER2-amplified breast tumors (16). It drives multiple aspects of tumor progression and metastasis by controlling the transcription of genes promoting cell proliferation and survival, genetic instability, differentiation block, cell invasion, and migration (13, 17). In addition, MYC maintains the tumor microenvironment by instructing tissue remodeling, angiogenesis, and inflammation, even when it is not overexpressed (18). In this context, MYC also controls the activation of tumor-associated macrophages, which increase cancer's aggressiveness (19), and regulates the expression of epithelial-to-mesenchymal transition (EMT) effectors (13). While most reports indicate that *MYC* is a prometastatic gene (20–27), some describe it as a metastatic suppressor instead (28–31), even suggesting that caution would be needed when designing clinical approaches against MYC in a metastatic setting.

Omomyc is a MYC dominant negative designed by our group, which comprises the MYC helix–loop–helix leucine zipper domain and harbors 4 amino acid substitutions in the leucine zipper that alter its dimerization specificity (32). Through extensive characterization, we have demonstrated that Omomyc prevents MYC's interaction with its natural partner MAX, sequestering it away from target promoters as Omomyc/MYC dimers and occupying the E-box consensus DNA-binding site in the form of inactive MAX/Omomyc and Omomyc/Omomyc dimers (33). Omomyc's antitumor activity as a genetically engineered transgene has been extensively described by our group and others against multiple tumor types (18, 34–40). Recently, the demonstration of the remarkable therapeutic impact elicited by the recombinantly produced Omomyc miniprotein in lung adenocarcinoma models constituted a paradigm shift, demonstrating that Omomyc could be used as a clinically viable drug (33, 41, 42). However, its therapeutic impact on metastatic disease has not been elucidated yet. Here we made use of a panel of breast cancer cell lines, representative of all molecular subtypes of the disease, to evaluate Omomyc's efficacy against breast cancer and metastasis. By inducible expression of the Omomyc transgene, we studied the effect of MYC inhibition on proliferation, angiogenesis, migration, and invasion. We also assessed Omomyc's impact *in vivo* on primary mammary tumor growth and, for the first time, on lung colonization and metastatic growth. In addition, to be able to translate these results to a pharmacologically meaningful context, we studied how exogenous administration of the Omomyc miniprotein compares with the expression of the Omomyc transgene, assessing its therapeutic effect in TNBC cell line- and patient-derived models.

## Materials and Methods

### Cell Lines and Lentiviral Infections

All cell lines were kindly provided by the laboratories of Violeta Serra [CAL-51 (DSMZ catalog no. ACC-302, RRID:CVCL_1110), JIMT-1 (DSMZ catalog no. ACC-589, RRID:CVCL_2077), MCF7 (ATCC catalog no. HTB-22, RRID:CVCL_0031), MDA-MB-361 (ATCC catalog no. HTB-27, RRID:CVCL_0620), MDA-MB-453 (ATCC catalog no. HTB-131, RRID:CVCL_0418), T-47D (ATCC catalog no. HTB-133, RRID:CVCL_0553), HCC1954 (ATCC catalog no. CRL-2338, RRID:CVCL_1259), and MCF 10A (ATCC catalog no. CRL-10317, RRID:CVCL_0598)], Joaquín Arribas [BT-474 (ATCC catalog no. HTB-20, RRID:CVCL_0179), MDA-MB-231 (ATCC catalog no. HTB-26, RRID:CVCL_0062), and SK-BR-3 (ATCC catalog no. HTB-30, RRID:CVCL_0033)], and Josep Villanueva [BT-549 (ATCC catalog no. HTB-122, RRID:CVCL_1092) and Hs 578T (ATCC catalog no. CRL-7849, RRID:CVCL_0332)] throughout 2013. The MDA-MB-231 cells used for isPLA were purchased from the ATCC repository in March 2021. CAL-51, Hs 578T, JIMT-1, and MCF7 cells were grown in DMEM (Life Technologies). BT-474, MDA-MB-231, SK-BR-3, and MCF 10A cells were grown in DMEM/F12 medium (Life Technologies). BT-549, T-47D, and HCC1954 cells were grown in RPMI medium (Life Technologies). MDA-MB-361 and MDA-MB-453 cells were grown in L-15 medium (Life Technologies). MCF 10A cells were supplemented with 5% horse serum, 20 ng/mL EGF, 0.5 μg/mL hydrocortisone, 100 ng/mL cholera toxin, 10 μg/mL insulin, 15 mmol/L HEPES, and 1% glutamine. All other cell lines were supplemented with 10% FBS and 1% glutamine. All cells were grown at 37°C and 5% CO_2_, except MDA-MB-361 and MDA-MB-453, which were grown without CO_2_. Cells were used for a maximum of 10 passages after collection or thawing. All cell lines were periodically checked for *Mycoplasma* contamination with the MycoAlert Assay Control Set (Lonza), following the manufacturer's instructions, and used only if the result was negative. MDA-MB-231 and BT-549 cell lines were authenticated at Instituto de Investigaciones Biomédicas “Alberto Sols” (CSIC-UAM) by microsatellite analysis.

Cell lines were infected with the pSLIK-Hygro lentiviral vector, a gift from Iain Fraser (RRID:Addgene_25737) containing a GFP or Omomyc cassette, which were inserted by gateway technology. For infections, HEK 293T cells (ATCC catalog no. CRL-3216, RRID:CVCL_0063) were seeded at 30% confluence, and the next morning 25 mmol/L chloroquine added. Two hours later, HEK 293T cells were transfected with pSLIK-Hygro-Omomyc or pSLIK-Hygro-GFP plus the lentiviral vectors pMD2.G (RRID:Addgene_12259) and psPAX2 (RRID:Addgene_12260) by the CaPO_4_ method. The medium was changed the next day and sodium butyrate added at 5 mmol/L. Viral supernatants were harvested on the subsequent 2 days, filtered with 0.45-μm polyvinylidene difluoride (PVDF) filters (Millipore) and added to target cells with polybrene (0.8 mg/mL). Transduced cells were selected with 1 mg/mL hygromycin.

For most *in vivo* experiments, MDA-MB-231 containing a pSLIK-Hygro-Omomyc vector obtained by lentiviral infection and a triple-fusion protein reporter construct encoding herpes simplex virus thymidine kinase 1, GFP, and firefly luciferase obtained by retroviral infection (43, 44) were used. In the biodistribution experiment, MDA-MB-231 cells containing a pQCXIH-firefly luciferase vector obtained by retroviral infection were used. In the experiment to study the effect of intravenous administration of the Omomyc miniprotein on primary tumor growth, wild-type MDA-MB-231 cells were used.

### Western Blots

For Western blots (WB), cells were cultured in the presence or absence of 0.6 μg/mL doxycycline for 3 days. Then, culture media was discarded and cells were placed on ice. PBS + 1 mmol/L EDTA was added to the plate and cells were scraped, centrifuged, and washed twice. Cell pellets were frozen and kept at −80°C.

Cells were lysed with RIPA buffer supplemented with protease and phosphatase inhibitors (Roche) and the protein fraction collected. Proteins were quantified by the DC Protein Assay (Bio-Rad) and absorbance at 560 nm was measured with a spectrophotometer (Victor3, PerkinElmer or TECAN Spark, Life Sciences). 10 to 30 μg of protein extract in Laemmli buffer +15% β-mercaptoethanol were run on 10% or 12% precast gels (Life Technologies). Proteins were transferred to nitrocellulose or PVDF membranes by the iBlot 2 Dry Blotting System (Life Technologies) and membranes were stained with Ponceau red. After washing with PBS + 0.1% Tween and blocking with 2% milk, membranes were incubated overnight at 4°C with the following primary antibodies: rabbit polyclonal anti-Omomyc (affinity purified and selected against recognition of the Myc B-HLH-LZ epitope, 1:5,000 or 1:12,000) rabbit monoclonal anti-CDK4 (D9G3E; Cell Signaling Technology, catalog no. 42749, RRID:AB_2799229, 1:1,000), rabbit polyclonal anti-Histone H3 (Abcam, catalog no. ab70550, RRID:AB_1209471, 1:500), and mouse monoclonal anti-β-actin (AC-15; Sigma-Aldrich, catalog no. A1978, RRID:AB_476692, 1:50,000). Membranes were washed with PBS-Tween and incubated for 1 hour at room temperature with the following secondary antibodies: ECL anti-rabbit IgG-HRP (GE Healthcare, catalog no. NA934, RRID:AB_772206, 1:5,000) and ECL anti-mouse IgG-HRP (GE Healthcare, catalog no. NA931, RRID:AB_772210, 1:5,000). Membranes were washed twice with PBS-Tween and once with PBS, and then incubated for 5 minutes with SuperSignal West Pico Chemiluminescent Substrate (Thermo Fisher Scientific) before revealing. To quantify the Omomyc levels expressed in each cell line, the intensity of the Omomyc and β-actin bands was quantified with ImageJ (RRID:SCR_003070; ref. 45) and a ratio Omomyc/β-actin established. This ratio was then correlated with the relative cell number obtained in the colony formation assays.

### Subcellular Fractionation

Subcellular fractionation was performed as previously described in ref. 46. 10 μg of protein from the core nuclear and cytoplasmic fractions were loaded and a WB performed. Therefore, only the lanes from the same fraction can be compared (nuclear vs. nuclear and cytoplasmic vs. cytoplasmic, but not nuclear vs. cytoplasmic), as the protein content does not reflect the relative amount obtained for each fraction. The perinuclear fraction is not shown due to the very low protein yield obtained.

### BrdU Incorporation and Cell-Cycle Analysis

For bromodeoxyuridine (BrdU) incorporation and cell-cycle analysis, MDA-MB-231-GFP, MDA-MB-231-Omomyc, or MDA-MB-231 cells were treated for 3 days with either vehicle, 0.6 μg/mL doxycycline or 20 μmol/L Omomyc, then labeled with 10 μmol/L BrdU for 2 hours, harvested by trypsinization, and fixed/permeabilized in absolute ethanol overnight at 4°C. After denaturation in 2 mol/L HCl for 30 minutes at room temperature, they were neutralized with 0.5 mol/L sodium borate and stained with anti-BrdU-FITC antibody (BD Biosciences, catalog no. 556028, RRID:AB_396304, 1:5) diluted in PBS + 0.5% BSA and 0.5% Tween. Cells were incubated in 25 mg/L propidium iodide (PI) + 100 μg/mL RNAse A and 0.3 μmol/L Triton X-100 for 30 minutes. Navios Flow Cytometer (Beckman Coulter) was used for data acquisition and FCS Express 4 software was used for data analysis.

### Proliferation Assay

20,000 MDA-MB-231-GFP or MDA-MB-231-Omomyc cells/well were plated in 6-well plates with or without 0.6 μg/mL doxycycline. After 6 days, cells were trypsinized and counted with the Tali Image-based Cytometer (Life Technologies).

### Clonogenic Assays

500 cells/well were plated in 6-well plates in the presence or absence of 0.6 μg/mL doxycycline. Cells were fixed with 4% formaldehyde between 2 and 7 weeks later, depending on the doubling time of each cell line. After 20 minutes of fixation, colonies were stained with 0.05% crystal violet for 1 hour and the plates washed with distilled water and air dried. The bottom of the plate was scanned in high resolution and the images obtained were analyzed with the ColonyArea plugin for ImageJ (47) to determine, for each well, the area percentage occupied with cells and the intensity of the staining, as described previously (47).

### Detection of Lentivirally Expressed Omomyc by Flow Cytometry

For the detection of lentivirally expressed Omomyc, MDA-MB-231-Omomyc cells were treated for 3 days with or without 0.6 μg/mL doxycycline, harvested by trypsinization and fixed/permeabilized in absolute ethanol overnight at 4°C. Cells were washed with PBS-Azide (PBS with 0.1% sodium azide and 1% BSA) and blocked with 2.5% BSA in PBS for 30 minutes at room temperature. Cells were stained with rabbit polyclonal anti-Omomyc antibody (1:50) for 1 hour at room temperature. After washing, cells were incubated with goat anti-rabbit AlexaFluor647 (1:1,000, Life Technologies) for 45 minutes at room temperature. Navios Flow Cytometer (Beckman Coulter) was used for data acquisition and FlowJo V10 software (RRID:SCR_008520) was used for data analysis.

### Angiogenesis Assay

1 million MDA-MB-231-GFP cells ± 0.6 μg/mL doxycycline, 1 million MDA-MB-231-Omomyc cells without doxycycline, and 1.5 million MDA-MB-231-Omomyc cells + 0.6 μg/mL doxycycline were plated and their conditioned media collected after 3 days. Cells expressing Omomyc were plated at a higher density so that at the time of medium collection, the number of cells was the same as the nonexpressing ones. Conditioned media was centrifuged and filtered with 0.45-μm PVDF filters (Millipore). 75,000 human umbilical vein endothelial cells (HUVEC) were mixed with 300 μL of each conditioned media and 100 μL of the mix containing 25,000 cells was plated per well in a 96-well plate, on top of a Matrigel layer to allow tube formation. After 6 hours, representative images were acquired with an IX71 Olympus inverted microscope. Tube formation was assessed by the Angiogenesis Analyzer plugin for ImageJ as described previously (48).

### Wound Healing Assay

MDA-MB-231-GFP and MDA-MB-231-Omomyc cells were plated with or without 0.6 μg/mL doxycycline. After 3 days, cells were trypsinized, counted, and 400,000 cells/well were seeded in triplicates in a 12-well plate ± doxycycline. Seven hours later, when cells were attached and forming a confluent monolayer, a p1000 pipette tip was used to scratch the surface of the well, forming a wound. An Olympus CellR microscope equipped with a Hamamatsu C9100 camera was used to follow the closure of the wound. Pictures were taken automatically every 30 minutes and the wound area was measured at the endpoint with ImageJ. Cells were then trypsinized, counted with the Tali Image-based Cytometer (Life Technologies) and the difference in wound area was corrected for the total number of cells.

### Boyden Chamber Assays

#### Migration Assay

MDA-MB-231-GFP, MDA-MB-231-Omomyc, BT-549-GFP, BT-549-Omomyc, CAL-51-GFP, CAL-51-Omomyc, MCF7-GFP, and MCF7-Omomyc cells were plated in complete medium (+10% FBS) with or without 0.6 μg/mL doxycycline. After 3 days, cells were washed with PBS, refed with medium containing 0.5% FBS ± doxycycline, and left overnight. Then, cells were counted with the Tali Image-based Cytometer (Life Technologies) and 30,000 cells/well were seeded on top of Corning FluoroBlok inserts in a 24-well plate. The inserts consisted of a chamber with a dark porous membrane (pore diameter: 8 μm). Cells were seeded in triplicates, ± doxycycline, in medium containing 0.5% FBS, and the bottom of the well was filled with complete medium ± doxycycline. After 24 or 48 hours, depending on the migratory capacity of each cell line, migrated cells were stained with the fluorescent CellTracker Green CMFDA dye (Life Technologies), fixed with 4% formaldehyde, and representative images were taken with an IX71 Olympus inverted microscope. In parallel, 30,000 cells/well were seeded in a 24-well control plate under the same conditions, with the exception of the inserts. After 24 or 48 hours, they were harvested by trypsinization and counted with the Tali Image-based Cytometer. Migrated cells from the acquired images were analyzed with Fiji (RRID:SCR_002285), a distribution of the ImageJ software (49). The values of the images from the same condition were averaged, normalized, and corrected for the difference in cell number obtained from the control plate, if any was observed.

#### Invasion Assay

MDA-MB-231-GFP and MDA-MB-231-Omomyc cells were plated under the same conditions as the migration assay. The only difference was the use of Corning BioCoat invasion chambers, in which the porous membrane is coated with a layer of Matrigel to mimic an ECM. Cells were allowed to invade through the coated membrane for 24 hours and they were fixed, stained, and counted as explained for the migration assay. A control plate under the same conditions was also plated, and the differences in invasive capacity of the cells were corrected for the difference in cell number.

### Immunofluorescence

Sections from paraffin-embedded tumors or lungs were cut at 3.5-μm thick. Antigen retrieval was performed by heating 20 minutes at 400 W in a microwave in a sodium citrate buffer (10 mmol/L sodium citrate, 0.05% Tween 20, pH 6.0). After blocking for 1 hour with serum-free Dako Protein Block (#X0909), slides were incubated overnight at 4°C with mouse monoclonal anti-Omomyc antibody (clone 21–1-3, Ximbio 153657, 1:100 dilution) combined with rabbit polyclonal anti-Ki67 (Abcam, catalog no. ab15580, RRID:AB_443209, 1:100) or with rabbit anti-cleaved caspase-3 (Cell Signaling Technology, catalog no. 9661, RRID:AB_2341188, 1:200) in Dako Ready-to-use diluent (S2022). After three times, TBS-Tween 20 washes, slides were incubated for 1 hour at room temperature with goat anti-mouse IgG (H+L)–AlexaFluor488 conjugate (Thermo Fisher Scientific A11001, 1:200), goat anti-rabbit IgG (H+L)–AlexaFluor594 conjugate (Thermo Fisher Scientific A11012, 1:200) and 5 μg/mL DAPI (Life Technologies D1306), washed three times with distilled water and mounted with fluorescence mounting medium (Dako S3023). Images were obtained with a Nikon Eclipse TI inverted epifluorescence microscope equipped with a Nikon DS-Qi2 camera.

### Omomyc Miniprotein Cell Entry by Flow Cytometry

MDA-MB-231 and MCF7 cells were treated with buffer (25 mmol/L NaAc 2 mol/L urea), 0.5 μmol/L, 1 μmol/L, or 5 μmol/L Omomyc-AlexaFluor 488 for 15 minutes. Then, they were trypsinized with 0.25% Trypsin-EDTA (Gibco) for 30 minutes at 37°C to remove membrane-bound peptide and trypsin was deactivated with 10% FBS. Cells were washed and resuspended in PBS and immediately analyzed. CytoFlex Flow Cytometer (Beckman Coulter) was used for data acquisition and CytExpert software (Beckman Coulter) was used for data analysis.

### *In Situ* Proximity Ligation Assay

MDA-MB-231 cells were seeded in 8-chamber tissue culture–treated glass slides (Falcon 354118) at cell density of 20,000 cells per chamber for 48 hours. Cells were then treated with either 20 μmol/L Omomyc or DMSO for 24 hours, after which culture medium was removed and cells were washed with 1× PBS at room temperature. Cells were fixed with 4% formaldehyde at room temperature for 10 minutes, then washed 2 times for 10 minutes with cold 1× PBS. Permeabilization was performed at room temperature using 1× PBS containing 0.2% Triton X100, 1% DMSO, and 1% blocking solution provided by the *in situ* proximity ligation assay (isPLA) Kit. Slides were washed three times for 5 minutes with 1× PBS at room temperature and incubated in blocking buffer (provided by the isPLA kit) for 1 hour at 37°C in a preheated humidity chamber. Incubation with primary antibodies was performed at 4°C overnight in the humidity chamber. The following antibodies were used: mouse monoclonal anti-MYC (G-4; Santa Cruz Biotechnology, catalog no. sc-373712, RRID:AB_10916994), rabbit polyclonal anti-MAX (Abcam, catalog no. ab101271, RRID:AB_10673343), rabbit polyclonal anti-Omomyc (affinity purified and selected against recognition of the Myc B-HLH-LZ epitope) and mouse monoclonal anti-Omomyc (clone 21–1-3, Ximbio 153657). isPLA was performed using the Duolink In Situ Detection Reagents Red (Sigma-Aldrich DUO92008) following the manufacturer's protocol using anti-rabbit plus (Sigma-Aldrich DUO92002) and anti-mouse minus (Sigma-Aldrich DUO92004) probes. DNA was stained with DAPI. Image acquisition was performed using ZEISS LSM 980 with Airyscan 2 confocal microscope (Carl Zeiss Microscopy GmbH) using the 20× objective. All image stacks were acquired with comparable settings, at a resolution of 2048 × 2048 pixels, z-stack size of 1 μm. Fluorescent dots were quantified using CellProfiler 4.2.1 software (RRID:SCR_007358; ref. 50). All data were analyzed using Rstudio (RRID:SCR_000432) version 4.1.0 with the following packages: tidyverse (RRID:SCR_019186) and ggplot2 (RRID:SCR_014601).

### Dose Response to the Omomyc Miniprotein

250–10,000 cells/well, depending on their size and growth rate, were seeded in 96-well plates and the next day treated with a single dose of increasing concentrations of Omomyc (0, 0.31, 0.63, 1.25, 2.5, 5, 10, 20, 40 and 80 μmol/L). After 5 days, alamarBlue (Thermo Fisher Scientific) was added to the wells and 4 hours later, fluorescence at 590 nm was measured with a Spark microplate reader (Tecan). GI_50_ values were calculated using GraphPad Prism 9 (RRID:SCR_002798).

### Combination with Paclitaxel

2,500 MDA-MB-231 and 1,000 BT-549 cells were seeded in 96-well plates and the next day treated with increasing concentrations of Omomyc (0, 2.5, 5, 10, 20 and 40 μmol/L) and/or paclitaxel (0, 0.1, 0.2, 0.39, 0.78, 1.56, 3.13, 6.25, 12.5 and 25 nmol/L). After 5 days, AlamarBlue (Thermo Fisher Scientific) was added to the wells and 2 hours later, fluorescence at 590 nm was measured with a Spark microplate reader (Tecan). ZIP synergy scores were calculated and plotted using SynergyFinder.org (51). When the synergy score ≤10, the interaction between two drugs is likely to be antagonistic; from −10 to 10, it is likely to be additive; and when >10, it is likely to be synergistic.

### Microarray Analysis

MDA-MB-231-Omomyc cells were seeded into 15 × 6 cm dishes, and 5 of them treated with 1 μg/mL doxycycline to induce the expression of Omomyc. The next day, 5 plates were treated with 20 μmol/L Omomyc and the remaining 5 with an equivalent volume of vehicle. After 3 days, plates were washed twice with PBS and frozen at −80°C until processing. The Omomyc miniprotein was added 1 day after doxycycline because our time-course experiments indicate that full expression of transgenic Omomyc takes around 12–24 hours. A higher number of cells were seeded on the +Omomyc plates compared with the control ones so that at collection, their number was equivalent. RNA was extracted with TRIzol reagent (Invitrogen) according to the manufacturer's instructions. The quality of RNA was confirmed with Agilent 2100 Bioanalyzer. Clariom S Human HT microarray plate (Applied Biosystems) was processed at Vall d'Hebron Institute of Research (VHIR)'s High Technology Unit. The microarray data were analyzed with Partek Genomics Suite software, v7.18.

Gene-set enrichment analysis (GSEA) was performed using publicly available software provided by the Broad Institute (RRID:SCR_003199, version 3.0) with the Hallmarks, Curated, Motif, GO, Oncogenic Signatures, and Immunological Signatures gene sets from the Molecular Signature Database (MSigDB; www.broad.mit.edu/gsea). We acknowledge our use of the GSEA software and MSigDB (www.broad.mit.edu/gsea; ref. 52). The number of permutations was set to 1,000, and the genes were ranked according to Signal2Noise.

### qRT-PCR

Quantitative reverse transcription polymerase chain reaction (qRT-PCR) was performed in MDA-MB-231-Omomyc cells treated with 1 μg/mL doxycycline for 4 days, 20 μmol/L Omomyc or vehicle for 3 days to mimic the microarray conditions. RNA was then extracted using RNeasy kit (QIAGEN) and quantified using NanoDrop. Equal amounts of RNA were reverse transcribed to generate cDNA using iScript Reverse Transcription Supermix for qRT-PCR (Bio-Rad). SYBR Green qRT-PCR analysis was then performed on these cDNA samples with PerfeCTa SYBR Green FastMix, Low Rox (Quantabio) using QuantStudio 6 FLEX system (Applied Biosystems). The data thus obtained were analyzed following the comparative (ΔΔ*C*_t_) method described in by Livak and Schmittgen (53). Glyceraldehyde 3-phosphate dehydrogenase (GAPDH) and β-Tubulin were used as housekeeping genes. Sequences of primers used are listed in Supplementary Table S2.

### cBioPortal for Cancer Genomics Data

We gathered genomic and clinical data from cBioPortal (54, 55) by performing a combined study on patient samples from four breast cancer datasets:

- Breast Invasive Carcinoma (TCGA, PanCancer Atlas; ref. 56)
- Breast Cancer (METABRIC ; refs. 57, 58)
- Breast Cancer (MSK 2018; refs. 59)
- Breast Cancer (MSK 2020; ref. 60)

We classified these samples according to the status of the seven selected genes significantly downregulated by Omomyc. Tumors bearing genetic alterations in at least 1 of the 7 seven genes were designated as “Altered” whereas the rest were considered “Unaltered.” We investigated differences in overall survival of “altered” versus “unaltered” patients and the frequency of genomic alterations in patients with amplification of at least 1 of the 7 genes (“amplified”) or without amplification (“unamplified”).

### Relapse-free Survival Plots

Relapse-free survival (RFS) plots were generated with the Kaplan–Meier Plotter for breast cancer tool (https://kmplot.com/), which contains clinical data from 7,830 patients with breast cancer (61).

### Animal Studies

All the animal studies were performed in accordance with the ARRIVE guidelines and the 3 Rs rule of Replacement, Reduction and Refinement principles. Mouse weights were recorded for every experiment. Mice were housed and treated following the protocols approved by the Ethical Committee for the Use of Experimental Animals (CEEA) at VHIR, Barcelona, Spain. The tumor sample from a patient used to establish the TNBC PDX was collected following an Institutional Research Board approved protocol and the associated written informed consent.

All the schematics were created with Biorender.com (RRID:SCR_018361).

#### Cell Line–Derived Orthotopic Model (Efficacy Studies)

##### Effect of Omomyc Expression on Primary Tumor Growth

a suspension of luciferase-expressing MDA-MB-231-Omomyc cells was mixed with Matrigel (1:1) and, after a small incision, 1.5 million cells/mouse were injected between the fourth and fifth right mammary fat pads of 22 6-week-old BALB/c nude female mice (Janvier RRID:IMSR_JCL:JCL:mID-0001). The wound was sutured with nonabsorbable thread (6–0). Before surgery, mice were anesthetized with 2% isoflurane and buprenorphine (0.75 mg/kg) was administered subcutaneously. Tumor size was evaluated three times a week by caliper measurements and tumor volume calculated using the following formula: volume = (length × width)^2^/2. When tumors reached 100 mm^3^, mice were randomized into two groups (*n* = 11). One group was given 2 g/L doxycycline in 5% sucrose in the drinking water to induce expression of the Omomyc transgene. The control group was given 5% sucrose. Sucrose is used to counteract the bitter taste of doxycycline so that mice drink enough water. Mice were treated for 4 weeks and euthanized by CO_2_ inhalation. Tumors were then excised, weighed, photographed, fixed for 48 hours in buffered 4% formaldehyde, transferred to 70% ethanol, and embedded in paraffin.

##### Effect of Omomyc Expression on Metastatic Growth

a suspension of luciferase-expressing MDA-MB-231-Omomyc cells were inoculated orthotopically into 50 six-week-old BALB/c nude females and tumor growth monitored as described above. Tumors were also followed by weekly IVIS imaging. Between 8 and 10 weeks after inoculation, mice were anesthetized by an intraperitoneal injection of a ketamine/xylazine mix (80 mg/kg ketamine, 10 mg/kg xylazine) and primary tumors were surgically resected. After resection, the 12 mice that presented metastases were randomized into two groups (*n* = 6) and treated with either 2 g/L doxycycline and 5% sucrose in the drinking water or with 5% sucrose only. The evolution of metastases was followed weekly by IVIS imaging, and mice were treated for a maximum of 12 weeks. Two control mice from the sucrose-treated group had to be euthanized 3 and 6 weeks after treatment onset due to metastatic burden. Mice were euthanized by CO_2_ inhalation, and an *ex vivo* IVIS scan was performed.

##### Effect of Intravenous Administration of the Omomyc Miniprotein on Primary Tumor Growth

a suspension of MDA-MB-231 cells was mixed with Matrigel (1:1) and 1.5 million cells/mouse were injected between the fourth and fifth right mammary fat pads of 13 six-week-old BALB/c nude females (Janvier). Before surgery, mice were anesthetized with 2% isoflurane and buprenorphine (0.75 mg/kg) was administered subcutaneously. Tumor size was evaluated once a week by caliper measurements, and tumor volume calculated using the following formula: volume = (length × width)^2^/2. When tumors were established, mice were sent to the Centro de Investigaciones Energéticas, Medioambientales y Tecnológicas (CIEMAT), their tumor volume analyzed by PET-CT, and randomized into two treatment groups. One group (*n* = 7) was treated intravenously with 50 mg/kg Omomyc twice a week, and the other group (*n* = 6) was treated with an equivalent amount of vehicle. Mice were treated for 4 weeks, a final PET-CT scan performed, and euthanized by CO_2_ inhalation. Caliper measures were also taken throughout the course of the treatment period.

#### Cell Line–Derived Lung Colonization Model (Efficacy Studies)

##### Effect of Omomyc Expression on Lung Colonization

a suspension of 500,000 luciferase-expressing MDA-MB-231-Omomyc cells in PBS were inoculated through the lateral tail vein of 20 BALB/c nude female mice (Janvier). Starting the day after inoculation, lung colonization and growth of tumor cells was monitored weekly by IVIS imaging. One week after inoculation, mice were randomized into two groups and treated with either 2 g/L doxycycline and 5% sucrose in the drinking water (*n* = 10) or with 5% sucrose only (*n* = 10). Five weeks later, a final *in vivo* bioluminescence detection was performed. Moreover, micro-CT (μCT) scans were performed to allow visualization of individual lung lesions. Mice were euthanized, and their lungs excised, fixed for 48 hours in buffered 4% formaldehyde, transferred to 70% ethanol and embedded in paraffin. Sections were cut 3.5-μm thick and stained with hematoxylin and eosin (H&E). Metastatic foci were counted and their area measured with ImageJ.

##### Effect of Pretreatment with the Omomyc Miniprotein on Lung Colonization

MDA-MB-231 cells were treated with 20 μmol/L Omomyc or an equivalent volume of vehicle and, after 3 days, trypsinized for 30 minutes with 0.25% trypsin to remove any potential membrane-bound Omomyc. 500,000 cells in PBS previously treated with vehicle were inoculated through the lateral tail vein of 15 BALB/c nude female mice (Janvier), and 500,000 cells in PBS previously treated with Omomyc were inoculated into 15 other female mice. After 24 days, mice were euthanized and their lungs excised, fixed for 48 hours in buffered 4% formaldehyde, transferred to 70% ethanol, and embedded in paraffin. Sections were cut 3.5-μm thick and stained with H&E. Whole lung sections were scanned with a digital slide scanner (Hamamatsu Photonics) and metastatic foci were counted and their area measured with the NDP.view2 viewing software.

#### Biodistribution Study

Omomyc was labeled with BDP-650/665 maleimide (Lumiprobe #58480) with an efficiency of 70%.

Twenty BALB/c nude female mice (Janvier) were used for the study. 10 mice received 0.5 million cells intravenously and 10 mice received 1.5 million cells orthotopically as described before to induce the formation of lung and mammary tumors, respectively. Lung tumor growth was followed by weekly IVIS imaging and mammary tumor growth was followed by caliper measurements. Seven weeks after inoculation, mice were treated with 50 mg/kg of Omomyc-BDP-650/665 and euthanized after 1 hour. Two mice from each model were treated with vehicle only and served as controls. Lungs and mammary fat pads were analyzed *ex vivo* by IVIS imaging and the signal emitted by labeled Omomyc was quantified.

#### Survival Study in a Patient-Derived Xenograft

A tumor biopsy obtained from a metastatic TNBC patient of the Vall d'Hebron University Hospital was expanded in NMRI nude female mice (Janvier RRID:IMSR_TAC:nmrinu), whose drinking water was supplemented with 1 μmol/L 17-β-estradiol. To obtain experimental mice, a tumor was excised, divided into 30 pieces of equal size, and each one inoculated subcutaneously into NMRI nude female mice. Briefly, mice were anesthetized with intraperitoneal ketamine/xylazine (80/10 mg/kg respectively). Methocel was administered for protection of eyes. After a small dorsolateral incision on the flank, a tumor piece was implanted and the wound closed with Histoacryl. Meloxicam (5 mg/kg) was administered subcutaneously on the day of the surgery and for 3 consecutive days. Tumor size was evaluated twice a week by caliper measurements and tumor volume calculated using the following formula: volume = (length × width)^2^/2. When tumors reached 100 mm^3^, mice were randomized into two groups. One group (*n* = 14) was given 50 mg/kg Omomyc intravenously twice a week and the other one (*n* = 15) was given an equal amount of vehicle twice a week. A survival study was performed and mice were treated until they showed excessive bodyweight loss (>20%), excessive tumor volume (>1,750 mm^3^) or tumor ulceration, and then were euthanized by CO_2_ inhalation.

#### IVIS, μCT, and PET-CT Imaging

IVIS studies were performed with a Xenogen IVIS Spectrum (Perkin Elmer). For imaging, mice were injected intraperitoneally with a d-luciferin solution (150 mg/kg in PBS) 5 to 10 minutes prior to acquisition. Mice were anesthetized with isoflurane (5% for induction and 2% during acquisition) and air flow was set at 0.8 L/minute. IVIS data were analyzed with Living Image software (Perkin Elmer). Study analysis consisted of a light radiance quantification. Signals from the light sources were detected and characterized. Working units were photons/sec/cm^2^/sr, which allow comparison between images obtained by different acquisition parameters. Acquisition and analysis were carried out by the Preclinical Imaging Platform staff at VHIR.

μCT studies were performed with a Quantum GX microCT Imaging System (PerkinElmer). Mice were anesthetized with isoflurane (5% for induction and 2% during acquisition) and air flow was set at 0.8 L/minute. μCT reconstructions were performed with the Quantum GX microCT software and AMIDE software was used for the analysis (http://amide.sourceforge.net, RRID:SCR_005940). Acquisition and analysis were carried out by the Preclinical Imaging Platform staff at VHIR.

PET-CT studies were performed using a small-animal PET scanner (SuperArgus PET-CT 4R, SEDECAL). Mice were fasted overnight prior to the PET-CT scan. PET (energy window 425–700 keV and 20-minute static acquisition in head-prone position) and CT studies (voltage 45 kV, current 150 μA, 8 shots, 360 projections, and standard resolution) were performed at 45 min after intravenous injection of ^18^FDG (8.6±0.9 MBq) via the tail vein in mice anesthetized by inhalation of 2%–2.5% isoflurane in 100% oxygen at a flow rate of 5% oxygen using a Fluovac System (Harvard Bioscience). Throughout the imaging session, body temperature was constantly kept at 38°C with a heating pad. PET images were corrected for random events and scatter with and without attenuation correction and reconstructed using the 3D–OSEM (Ordered Subset Expectation Maximization) algorithm (16 subsets and 3 iterations). Free-hand regions-of-interest (ROI) were drawn along the tumors in the CT images; ^18^FDG PET images were used to help locate and delimit the tumor in the CT. Images were analyzed using the image analysis software AMIDE, version 1.0.4. Acquisition and analysis were carried out at CIEMAT.

### Statistical Analysis

For *in vitro* experiments, a minimum of three biological replicates with 1–3 technical replicates was used. For *in vivo* experiments, the number of mice used per treatment group is indicated in the text.

Statistical analysis and representation of the data was performed using GraphPad Prism 9. For histograms, mean + SD or mean – SD is shown if the mean is positive or negative, respectively. For scattered dot plots, mean ± SD is shown. For X-Y graphs, mean ± SD, mean + SD or mean − SD is shown depending on the overlap between groups to help data visualization, unless otherwise specified.

To determine statistical significance among two groups, a two-tailed unpaired *t* test (parametric) or a two-tailed Mann–Whitney test (nonparametric) was used. Outliers were identified with the ROUT method and discarded from the analysis and graphical representation.

For all tests, an alpha level of 0.05 was established. In all graphs, *, **, *** and **** are used to describe *P* values below 0.05, 0.01, 0.001, and 0.0001, respectively. For all histograms and scattered dot plots, asterisks above one bar/dot plot indicate statistical significance between that group and the control group.

### Data Availability Statement

The data generated in this study are available within the article and its Supplementary Data files. Microarray data are publicly available in Gene Expression Omnibus (GEO) (RRID:SCR_005012) at GSE174447.

## Results

### Expression of Omomyc in Breast Cancer Cells Alters Their Cell Cycle and Reduces Proliferation

To test the hypothesis that Omomyc is an effective therapy against breast cancer, we infected a panel of human breast cancer cell lines with lentiviral vectors containing a doxycycline-inducible Omomyc cassette or a GFP control. Eleven stable cell lines representative of the four intrinsic molecular subtypes of breast cancer were generated. We specifically focused on TNBC, the subtype with fewer therapeutic options. In particular, we made use of the well-characterized *TP53*-, *BRAF*- and *CDKN2A*-mutated MDA-MB-231 cell line as a paradigm metastatic TNBC model for most *in vitro* and *in vivo* studies. Doxycycline-dependent expression of Omomyc in most cell lines was confirmed by WB after a 3-day doxycycline treatment (Supplementary Fig. S1A), and, in the case of MDA-MB-231, confirmed by flow cytometry as well (Supplementary Fig. S1B).

**FIGURE 1:**
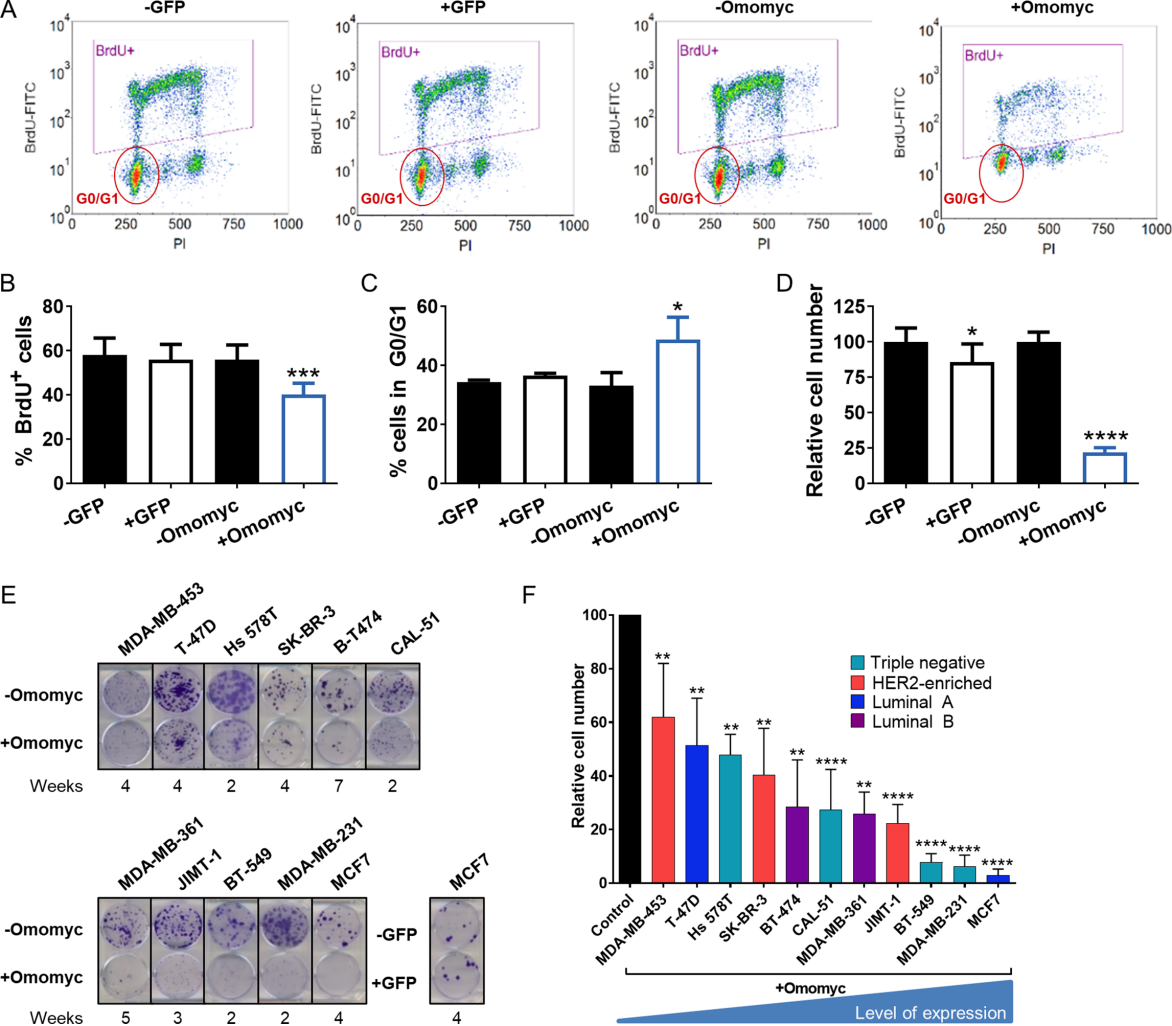
Expression of Omomyc in breast cancer cells alters their cell cycle and reduces proliferation. **A,** Representative density plots of MDA-MB-231-GFP untreated cells (−GFP) or treated with 0.6 μg/mL doxycycline (+GFP) and MDA-MB-231-Omomyc untreated cells (−Omomyc) or treated with 0.6 μg/mL doxycycline (+Omomyc) for 3 days measured by flow cytometry. BrdU-FITC: Bromodeoxyuridine-Fluorescein isothiocyanate. PI, propidium iodide. **B,** Percent of BrdU-positive MDA-MB-231 cells from **A**. Graph shows mean + SD; statistical significance was determined by two-tailed unpaired *t* test. **C,** Percent of G_0_–G_1_ MDA-MB-231 cells from **A**. Graph shows mean + SD; statistical significance was determined by two-tailed unpaired *t* test. **D,** Cell counts of MDA-MB-231-GFP and MDA-MB-231-Omomyc cells treated with doxycycline for 6 days. Graph shows mean + SD; statistical significance was determined by two-tailed unpaired *t* test. **E,** Representative images of wells stained with crystal violet after Omomyc expression in a panel of breast cancer cell lines. An example of a GFP-expressing cell line is also shown. Expression of Omomyc or GFP was induced by addition 0.6 μg/mL doxycycline in the culture media. **F,** Quantification of relative cell number after Omomyc expression compared with control cell without doxycycline. Graph shows mean + SD; statistical significance was determined by two-tailed Mann–Whitney test.

We then characterized the cell cycle of MDA-MB-231 cells expressing GFP (MDA-MB-231-GFP) or Omomyc (MDA-MB-231-Omomyc) upon 3-day doxycycline treatment (Fig. 1A). In these assays, Omomyc expression clearly reduced DNA synthesis during the S-phase of the cell cycle (Fig. 1A and B) and caused accumulation of cells in the G_0_–G_1_ phase, while control GFP had no detectable effect (Fig. 1A–C). This alteration in cell cycle dramatically decreased the cell number after 6 days of Omomyc treatment, while cells expressing GFP showed only a slight reduction (Fig. 1D).

To confirm whether the impact of MYC inhibition was conserved across different genetic backgrounds and breast cancer subtypes, our panel of cell lines was seeded at low density and treated for periods of 2 to 7 weeks (depending on their proliferation rate), and then stained with crystal violet. While cells expressing GFP showed no impairment in their colony formation capacity, all Omomyc-expressing cells presented fewer and smaller colonies (Fig. 1E). Quantification of the crystal violet area and intensity (a readout of cell number) revealed significant reductions when Omomyc (Fig. 1F), but not GFP (Supplementary Fig. S1C), was expressed in each cell line. The degree of sensitivity to Omomyc expression was variable among cell lines and did not correlate with molecular subtype, MYC levels, or the extent of MYC downregulation, whereas it showed an inverse correlation with Omomyc expression levels (Supplementary Fig. S1D). Hence, the variability in the response to Omomyc is not due to intrinsic genetic differences among cell lines but rather to the amount of transgenic Omomyc that the cells are expressing (Fig. 1F).

### Omomyc Impairs the Capacity of MDA-MB-231 Cells to Induce Angiogenesis *In Vitro*

**FIGURE 2:**
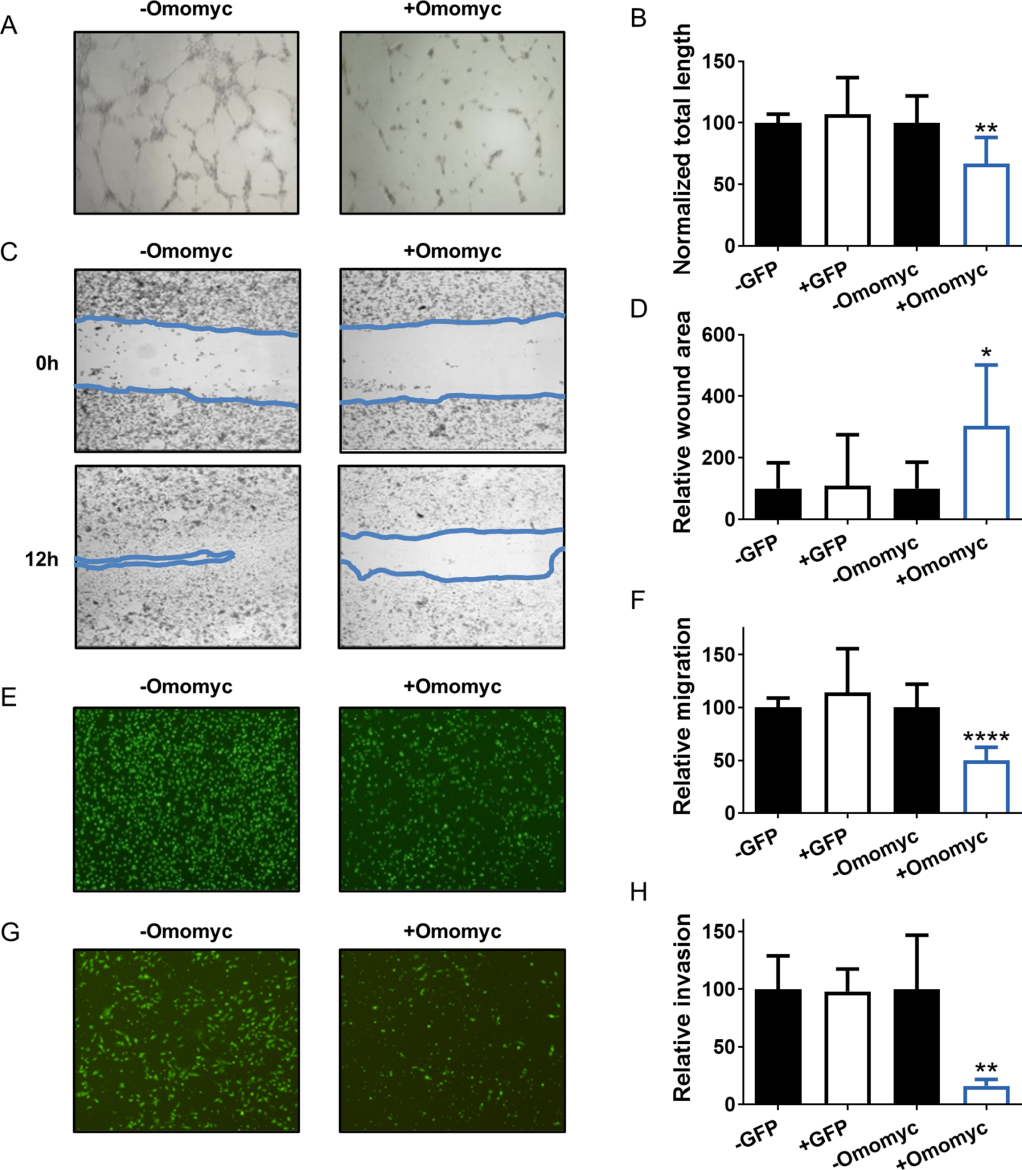
Omomyc impairs the capacity of MDA-MB-231 cells to induce angiogenesis and reduces their metastatic potential *in vitro*. **A,** Representative images of HUVECs 6 hours after addition of conditioned media from MDA-MB-231-Omomyc cells untreated (−Omomyc) or treated for 3 days with 0.6 μg/mL doxycycline (+Omomyc). **B,** Quantification of total tube length in HUVECs from **A** and in HUVEC cells treated with conditioned media from control MDA-MB-231-GFP cells under the same conditions. Graph shows mean + SD; statistical significance was determined by two-tailed unpaired *t* test. **C,** Representative images of a wound-healing assay of MDA-MB-231-Omomyc cells at 0 hours (onset) and 12 hours (endpoint) after treatment with 0.6 μg/mL doxycycline (+Omomyc) or left untreated (−Omomyc). The approximate area of the wound is delimited by a blue line. **D,** Quantification of the relative wound area from **C** at endpoint and from control MDA-MB-231-GFP cells under the same conditions, corrected for the total cell number. Graph shows mean + SD; statistical significance was determined by two-tailed Mann–Whitney test. **E,** Representative images of fluorescently labeled MDA-MB-231-Omomyc cells after 24 hours of migration through and FBS gradient. Cells were plated on top of a Boyden chamber in 0.5% FBS with (+Omomyc) or without (−Omomyc) 0.6 μg/mL doxycycline and migrated through a porous membrane toward wells containing 10% FBS. **F,** Quantification of migrated cells from **E** and from control MDA-MB-231-GFP cells under the same conditions, corrected for the total cell number. Graph shows mean + SD; statistical significance was determined by two-tailed unpaired *t* test. **G,** Representative images of fluorescently labeled MDA-MB-231-Omomyc cells after 24 hours of invasion through and FBS gradient. Cells were plated on top of a Matrigel-coated Boyden chamber in 0.5% FBS with (+Omomyc) or without (−Omomyc) 0.6 μg/mL doxycycline and migrated through a porous membrane toward wells containing 10% FBS. **H,** Quantification of invaded cells from **G** and from control MDA-MB-231-GFP cells under the same conditions, corrected for the total cell number. Graph shows mean + SD; statistical significance was determined by two-tailed unpaired *t* test.

Given that MYC is a recognized proangiogenic factor (62) and that angiogenesis is a key aspect in tumor growth, survival, and metastasis (63), we analyzed the capacity of MDA-MB-231 cells to induce angiogenesis after 3 days of Omomyc expression and compared it with that of untreated or GFP-expressing cells. HUVECs exposed to conditioned media from Omomyc-expressing TNBC cells presented a clear impairment in tube formation after only 6 hours (Fig. 2A and B; Supplementary Fig. S2A–S2F), demonstrating that Omomyc expression is antiangiogenic *in vitro*.

### Omomyc Reduces the Metastatic Potential of Breast Cancer Cells *In Vitro*

To test the capacity of Omomyc to affect the metastatic phenotype of TNBC cells, we conducted migration and invasion assays in MDA-MB-231-GFP and MDA-MB-231-Omomyc cells. In a wound-healing assay, 12 hours of Omomyc expression led to a significant delay in the gap closure (Fig. 2C and D). Furthermore, in a Boyden chamber migration assay, Omomyc reduced cell migration toward nutrients by 50% after 24 hours (Fig. 2E and F). Finally, to test the invasive capacity of cells to migrate through an extracellular matrix (ECM), we made use of a Boyden chamber assay where cells have to migrate through a layer of Matrigel that mimics the ECM (64) and the process by which metastatic cells reach the bloodstream or the lymphatic system in the invasion process *in vivo*. Under these conditions, Omomyc-expressing cells showed a dramatic (80%) reduction in their invasive capacity (Fig. 2G and H). Taken together, these results demonstrate that Omomyc can impair several aspects of the metastatic phenotype of MDA-MB-231 cells.

To verify whether this was also true for other breast cancer cell lines besides MDA-MB-231, Boyden chamber migration experiments were performed in an additional highly metastatic cell line (BT-549), and two poorly metastatic cell lines (MCF7 and CAL-51). Even though the intrinsic ability of the cells to migrate was very different among cell lines, in all cases, Omomyc expression reduced their migratory capacity by approximately 50% (Supplementary Fig. S2G–S2I).

### Omomyc Expression Reduces the Growth of Primary Mammary Tumors *In Vivo* in a Cell Line–Derived Orthotopic Model

**FIGURE 3:**
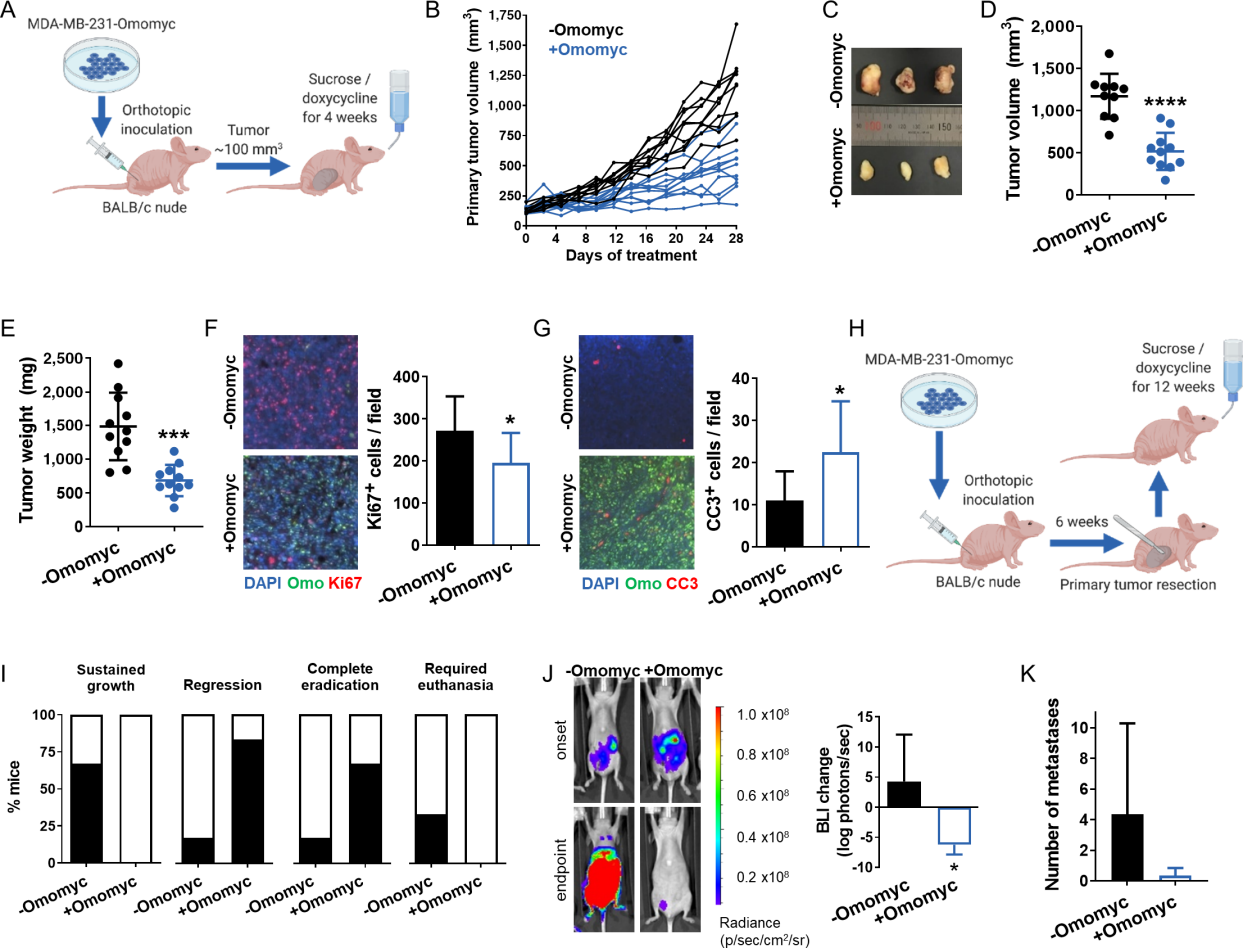
Omomyc expression reduces primary mammary tumor growth and causes regression of established metastases in a cell line–derived orthotopic model. **A,** Schematic representation of the mouse model used to assess primary tumor growth. Luciferase-expressing MDA-MB-231-Omomyc cells were inoculated into BALB/c nude females. When tumors reached 100 mm^3^, mice were treated with 5% sucrose (−Omomyc) or with 2 g/L doxycycline in 5% sucrose (+Omomyc) for 4 weeks. **B,** Tumor volume of sucrose- and doxycycline-treated tumors for 4 weeks. **C,** Image of three representative tumors from sucrose- and doxycycline-treated mice. **D,** Tumor volume at experimental endpoint. Graph shows mean ± SD; statistical significance was determined by two-tailed Mann–Whitney test. **E,***Ex vivo* tumor weight at experimental endpoint. Graph shows mean ± SD; statistical significance was determined by two-tailed unpaired *t* test. **F,** Representative images of double immunofluorescence for Omomyc (Omo) and Ki67 in control and treated tumors with its quantification. Graph shows mean + SD; statistical significance was determined by two-tailed unpaired *t* test. **G,** Representative images of double immunofluorescence for Omomyc (Omo) and cleaved caspase-3 (CC3) in control and treated tumors with its quantification. Graph shows mean + SD; statistical significance was determined by two-tailed unpaired *t* test. **H,** Schematic representation of the mouse model used to assess metastatic growth. Luciferase-expressing MDA-MB-231-Omomyc cells were inoculated into BALB/c nude females. After 8–10 weeks, primary tumors were surgically resected and mice were treated with 5% sucrose (−Omomyc) or with 2 g/L doxycycline in 5% sucrose (+Omomyc) for 12 weeks. **I,** The percentage of mice from each treatment group showing sustained growth, regression, and eradication of metastasis, as well as the percentage of mice that required euthanasia due to excessive metastatic growth. **J,** IVIS-acquired images of a representative sucrose-treated and a doxycycline-treated mouse at treatment onset and endpoint, and quantification of BLI. Graph shows mean + SD (−Omomyc) and mean − SD (+Omomyc); statistical significance was determined by two-tailed unpaired *t* test. **K,** Number of organs with metastasis per mouse in each treatment group. Graph shows mean + SD; statistical significance was determined by two-tailed unpaired *t* test.

To test the effect of Omomyc in TNBC *in vivo*, luciferase-expressing MDA-MB-231–Omomyc cells were inoculated orthotopically in the mammary fat pad of BALB/c nude mice. When tumors reached 100 mm^3^, mice were treated with 5% sucrose or 2 g/L doxycycline diluted in 5% sucrose added to their drinking water for 4 weeks (schematic in Fig. 3A). Tumors from Omomyc-expressing mice showed a much slower growth rate than controls (Fig. 3B) that translated into a very significant difference in tumor volume and tumor weight at experimental endpoint (Fig. 3C–E). At that stage, most of the control animals were close to the ethical endpoint in terms of tumor volume, while none of the treated ones had reached that stage yet (Fig. 3B). Notably, Omomyc expression did not cause any weight loss in the animals throughout the experiment, except for a drop during the first 2 days that was reverted at 4 days and was probably due to the change to water supplemented with doxycycline. In fact, differences observed in mouse weight from 9 days onwards were mainly due to differences in tumor size (Supplementary Fig. S3A). To check for Omomyc's effect at the cellular level, double immunofluorescence (IF) for Omomyc and the proliferation marker Ki67 or the apoptotic marker cleaved caspase-3 (CC3) was performed in tumor sections of control and treated mice. As expected, Omomyc expression impaired tumor growth by both decreasing cell proliferation (Fig. 3F) and enhancing apoptosis (Fig. 3G).

### Omomyc Expression Causes Regression of Established Metastases After Primary Tumor Resection

Following these encouraging results, we further tested Omomyc's effect against metastasis *in vivo*, in a model in which cells metastasize from the primary tumor site. Luciferase-expressing MDA-MB-231–Omomyc cells were inoculated orthotopically in the mammary fat pad of BALB/c nude mice until primary tumors established. Then, these were surgically resected to mimic the standard clinical procedure for human patients, which, unfortunately, is often followed by metastatic growth in secondary sites. After surgery, metastasis-bearing mice were treated with sucrose or doxycycline, and metastatic growth followed for 12 weeks (schematic in Fig. 3H). The majority of sucrose-treated mice showed sustained growth of established metastases, while most of doxycycline-treated mice actually showed metastatic regression and even complete eradication (Fig. 3I and J). Indeed, the difference in bioluminescence intensity (BLI) from treatment onset until experimental endpoint was calculated for both groups and was significantly different. The mean BLI change was positive in the control group, indicating metastatic growth, and negative in the treated one, indicating metastatic regression (Fig. 3J, right). Most tellingly, after *ex vivo* analysis of the mice, control animals presented an average of 4.3 organs with metastasis, compared with 0.3 in treated mice (Fig. 3K).

### Omomyc Expression Reduces Metastatic Lung Colonization

**FIGURE 4:**
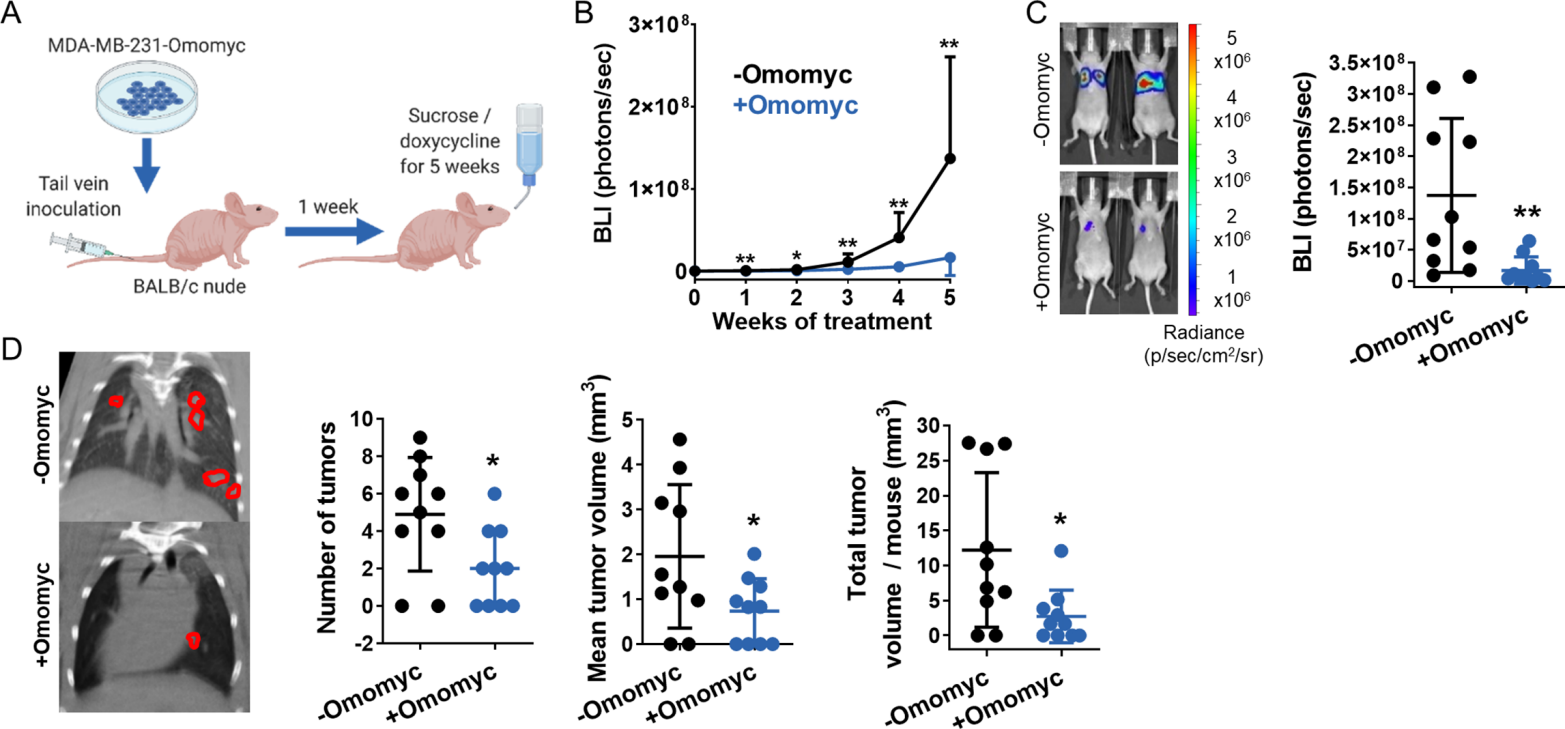
Omomyc expression reduces tumor appearance and growth in a cell line–derived model of lung colonization. **A,** Schematic representation of the mouse model. Luciferase-expressing MDA-MB-231 cells were inoculated through the lateral tail vein into BALB/c nude females. 1 week later, they were treated with 5% sucrose (−Omomyc) or with 2 g/L doxycycline in 5% sucrose (+Omomyc) for 5 weeks. **B,** Quantification of luciferase activity measured weekly by IVIS imaging as BLI intensity over the 5-week treatment. Graph shows mean + SD (−Omomyc) and mean − SD (+Omomyc); statistical significance was determined via two-tailed Mann–Whitney test. **C,** IVIS-acquired images of two representative sucrose-treated and doxycycline-treated mice and quantification of BLI intensity at experimental endpoint. Graph shows mean ± SD; statistical significance was determined via two-tailed Mann–Whitney test. **D,** Representative μCT images of the thoracic cavity of 3 mice treated with sucrose and 3 mice treated with doxycycline at experimental endpoint, with lung tumors circled in red (left), and quantification of the number of tumors, mean tumor volume and total tumor volume per mouse (right). Graphs show mean ± SD; statistical significance was determined via two-tailed unpaired *t* test (number of tumors and mean tumor volume) or via two-tailed Mann–Whitney test (total tumor volume per mouse).

After demonstrating how Omomyc impacts established metastases, we wondered whether its expression would also prevent them in the context of lung colonization, an earlier stage related to metastatic seeding and initial growth. Luciferase-expressing MDA-MB-231-Omomyc cells were injected into the bloodstream of BALB/c nude mice through the lateral tail vein, and, 1 week later, animals received sucrose or doxycycline for five consecutive weeks (schematic in Fig. 4A). Strikingly, lung lesions in doxycycline-treated mice presented hardly any growth compared with control mice, showing statistical differences in BLI intensity already 1 week after treatment onset, which were maintained until the endpoint (Fig. 4B and C). To analyze lung lesions individually, μCT images of the thoracic cavity were acquired for each mouse after 5 weeks of treatment. Lung tumors were counted and their volume calculated, showing that Omomyc expression significantly reduced both their number and volume, resulting in a significant decrease of the total tumor burden per mouse (Fig. 4D). As in primary tumors, Omomyc expression significantly reduced cell proliferation (Supplementary Fig. S4A).

In addition, given that the resolution of μCT does not allow the detection of micrometastases, lung sections from untreated and treated mice were stained with hematoxylin and eosin (H&E) and their number and area calculated. As expected, lungs from Omomyc-treated mice presented smaller tumors (Supplementary Fig. S4B and S4C), but also a lower number of them (including micrometastases; Supplementary Fig. S4D), suggesting that Omomyc is not only affecting their growth but also the successful establishment of micrometastases after seeding.

### The Omomyc Miniprotein as a Pharmacologic Approach to Inhibit MYC in Breast Cancer

To translate the encouraging data obtained with the Omomyc transgene into a viable clinical option for patients with breast cancer, we made use of the recombinantly produced Omomyc miniprotein, an 11-kDa drug that displays cell-penetrating properties, reaches the nuclei, and has already shown remarkable antitumor activity in non–small cell lung cancer (NSCLC) models *in vitro* and *in vivo* (41). This protein is currently being tested in clinical trials in solid tumors (ClinicalTrials.gov identifier NCT04808362).

**FIGURE 5:**
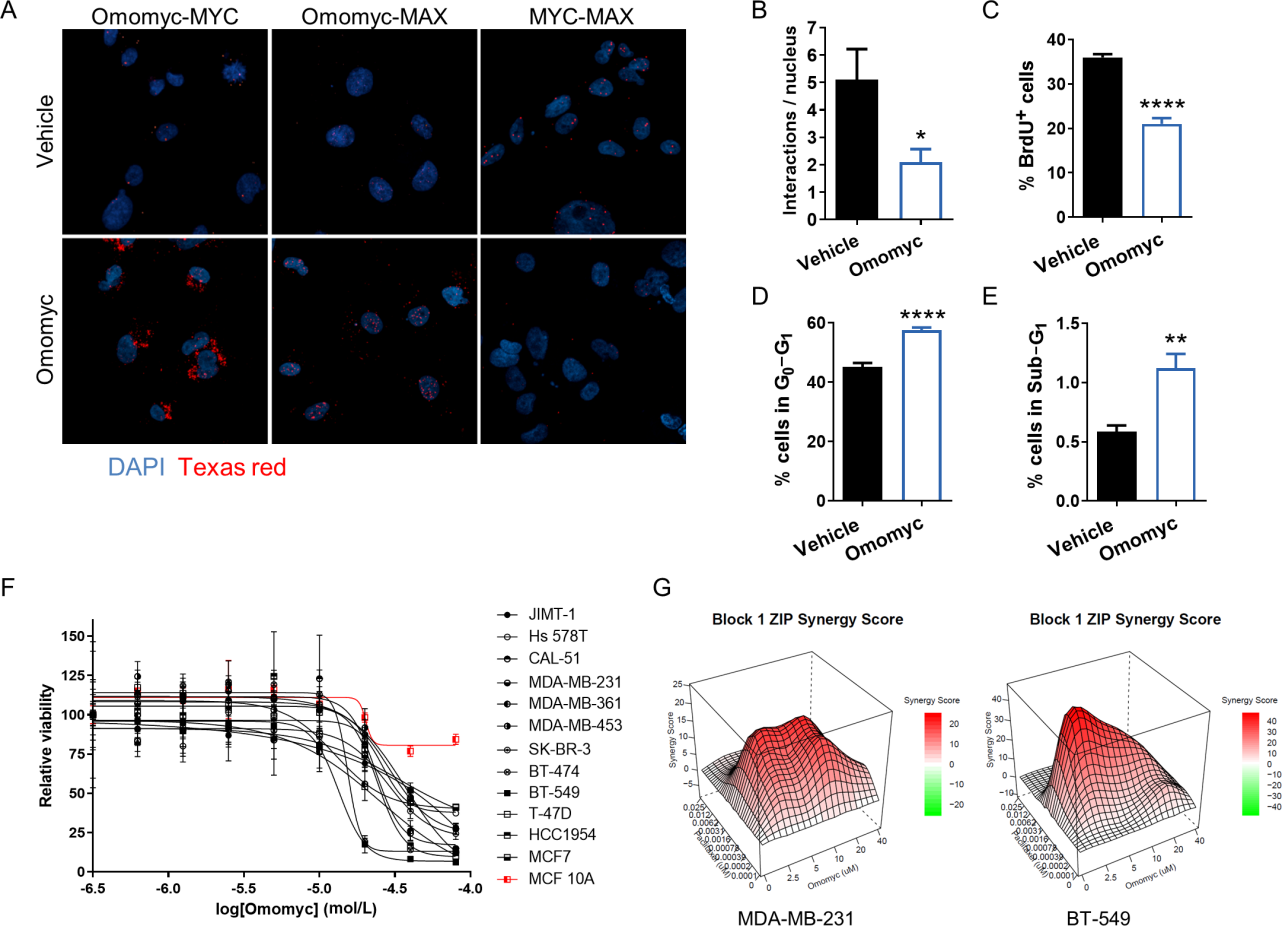
The Omomyc miniprotein binds to MYC and MAX, reduces the number of MYC–MAX interactions, has an impact on the cell cycle and reduces cell viability, alone and in combination with paclitaxel. **A,** isPLA of Omomyc-MYC, Omomyc-MAX, and MYC–MAX interactions after 24 hours of treatment with vehicle (DMSO) or 20 μmol/L Omomyc. Each red dot corresponds to an interaction. **B,** Number of MYC–MAX interactions from **A**. Graph shows mean + SD; statistical significance was determined via two-tailed unpaired *t* test. **C–E,** Percent of MDA-MB-231 cells positive for BrdU (**C**), in the sub-G_1_ phase of the cell cycle (**D**), or in the G_0_–G_1_ phase (**E**) after treatment with PBS (vehicle) or 20 μmol/L Omomyc for 3 days, measured by flow cytometry. Graphs show mean + SD; statistical significance was determined via two-tailed unpaired *t* test. **F,** Cell viability in a panel of human breast cancer cell lines and in the nontransformed mammary MCF 10A cells treated with increasing concentrations of Omomyc for 5 days. Graph shows mean ± SD. **G,** Graphical representations of the synergy score of MDA-MB-231 and BT-549 TNBC cells treated with increasing concentrations of paclitaxel and Omomyc, alone and in combination, for 5 days.

We treated MDA-MB-231 and MCF7 cells with increasing concentrations of fluorescently labeled Omomyc and observed that, within 15 minutes, the miniprotein was internalized in a dose-dependent manner, demonstrating for the first time its spontaneous cell-penetrating capacity in breast cancer (Supplementary Fig. S5). To confirm that the Omomyc miniprotein was functional inside the cells, MDA-MB-231 cells were treated with vehicle or 20 μmol/L Omomyc and, after 24 hours, the Omomyc-MYC, Omomyc-MAX, and MYC–MAX interactions were imaged by isPLA (Fig. 5A). As expected, Omomyc interacts with both MYC and MAX, and causes a reduction in the number of MYC–MAX dimers (Fig. 5A and B). Interestingly, most Omomyc-MAX dimers colocalize with DNA, while most Omomyc-MYC dimers do not.

To characterize the effect of the Omomyc miniprotein on TNBC proliferation, MDA-MB-231 cells were incubated with 20 μmol/L Omomyc or an equivalent volume of vehicle for 3 days, and their cell cycle analyzed. The cell-cycle profile was similar to the one previously shown with the Omomyc transgene (Fig. 1B–D), with a reduction of DNA incorporation during the S-phase and an increase in the percentage of cells in G_0_–G_1_ (Fig. 5C and D). In addition, Omomyc-treated cells also showed an increase in the sub-G_1_ population, indicating that the miniprotein is capable of inducing cell death (Fig. 5E). We then tested increasing concentrations of the Omomyc miniprotein in other breast cancer cell lines, observing a dose-dependent reduction in viability for all of them, with GI_50_ values in the low micromolar range (Fig. 5F). Interestingly, nontransformed mammary MCF 10A cells were comparatively more resistant to the treatment, showing only a slight reduction in cell number at very high concentrations of Omomyc (Fig. 5F). This is indicative of MYC dependence exclusively in aggressive tumor cell lines.

TNBC is currently treated mainly with chemotherapy (e.g., anthracyclines and taxanes) in the neoadjuvant and adjuvant setting (65). Importantly, aberrant MYC expression has been described to induce resistance to chemotherapy in general, and paclitaxel in particular, in breast and other cancers (66, 67). For this reason, we explored the therapeutic effect of the combination of Omomyc with paclitaxel in TNBC. To do so, we treated metastatic TNBC cell lines MDA-MB-231 and BT-549 with increasing concentrations of paclitaxel and Omomyc, alone or in combination, for 5 days. In both cell lines, the combination was more potent than either standalone therapy and showed synergy particularly at lower concentrations (Fig. 5G), indicating that the combination could show increased efficacy *in vivo*, or that the concentration of both drugs could be reduced to obtain the same efficacy with potential lower toxicity.

**FIGURE 6:**
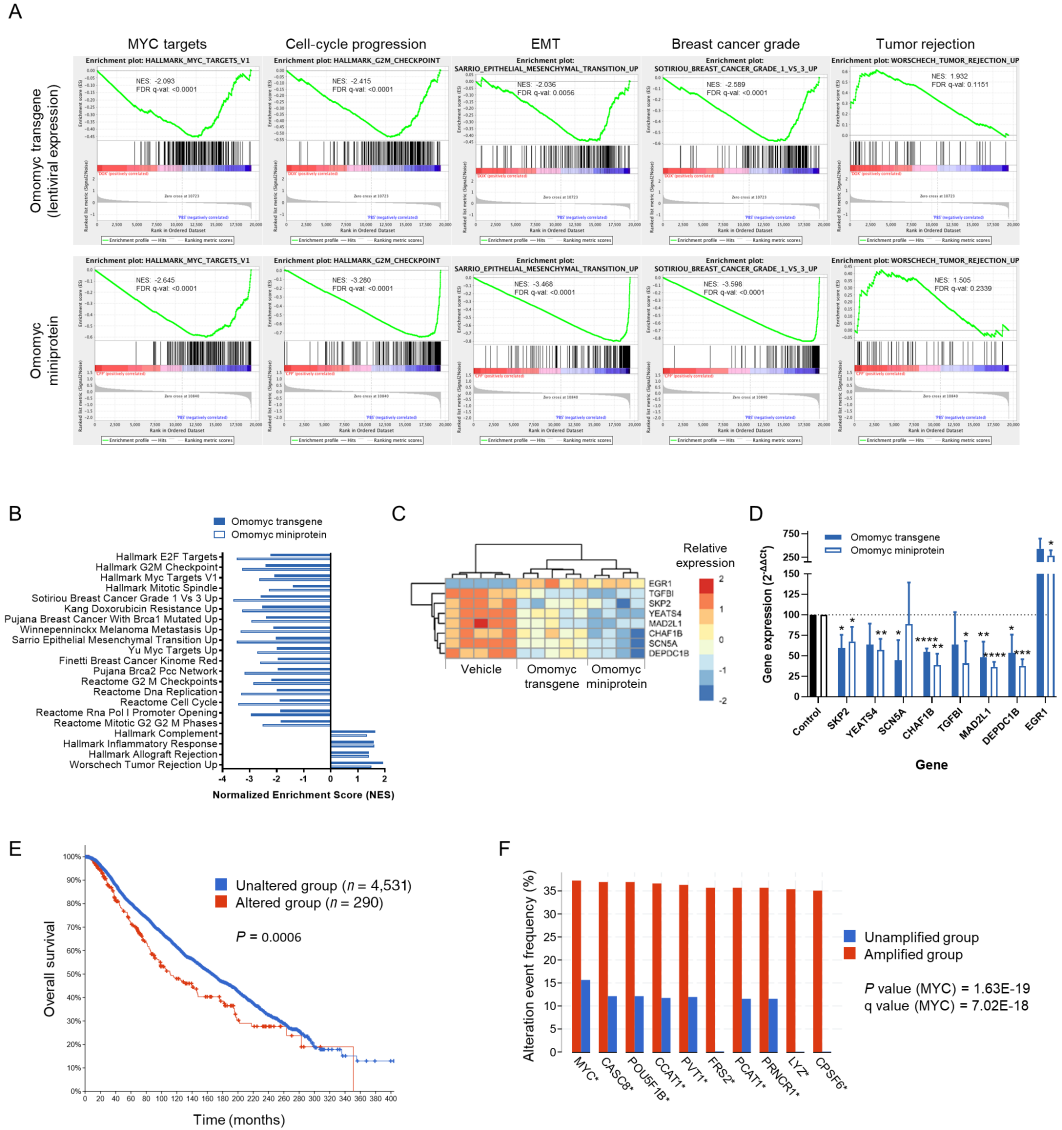
Lentiviral expression of an Omomyc construct (1 μg/mL doxycycline for 4 days) or treatment with the Omomyc miniprotein (20 μmol/L for 3 days) has a profound impact on MDA-MB-231 gene expression. **A,** Gene set enrichment analysis (GSEA) of selected cancer-related gene sets in MDA-MB-231-Omomyc cells that are significantly regulated by both conditions. Normalized Enrichment score (NES) and FDR q value (q-val) are shown. PBS, vehicle; DOX, Omomyc transgene; CPP, Omomyc miniprotein. **B,** NES of a selection of gene sets implicated in several hallmarks of breast cancer, that are significantly regulated by both conditions. The cutoff FDR q-val was set at 0.25. **C,** Relative expression by microarray analysis of a selection of genes significantly regulated by both conditions. **D,** Quantification of the mRNA expression by qRT-PCR from genes shown in **C** under the same treatment conditions. Graph shows mean + SD; statistical significance was determined via two-tailed unpaired *t* test. **E,** Kaplan–Meier overall survival plot of patients with breast cancer harboring genomic alterations in at least 1 out of the 7 genes downregulated by Omomyc shown in **C** (*TGFBI*, *SKP2*, *YEATS4*, *MAD2L1*, *CHAF1B*, *SCN5A*, and *DEPDC1B*). Source: cBioPortal. **F,** Top 10 altered genes of patients with breast cancer harboring genomic amplifications in at least 1 of the 7 genes downregulated by Omomyc shown in **C** (*TGFBI*, *SKP2*, *YEATS4*, *MAD2L1*, *CHAF1B*, *SCN5A*, and *DEPDC1B*). Source: cBioPortal.

To get more insight into Omomyc's mechanism of action against all breast cancer cell lines tested, we performed microarray analysis. For this analysis, we made use of MDA-MB-231-Omomyc cells treated with either doxycycline for 4 days to activate transgenic Omomyc expression or with 20 μmol/L of Omomyc miniprotein for 3 days. The analysis showed that the transgene and the miniprotein significantly modulated gene sets that confirm shutdown of the MYC fingerprint (downregulation of MYC targets) and can explain its antitumor and antimetastatic activity observed *in vitro* and *in vivo* (downregulation of cell-cycle progression, EMT and breast cancer grade, and upregulation of tumor rejection genes, among many others; Fig. 6A and B). Interestingly, the Omomyc miniprotein regulated more genes and gene sets than its transgenic counterpart (Supplementary Fig. S6A and S6B), probably due to higher nuclear levels (Supplementary Fig. S6C). At the individual gene level, we observed a certain overlap between the transgene and the miniprotein. In particular, 22.7% of the downregulated and 15.5% of the upregulated genes by the transgene were also regulated by the miniprotein. At the gene set level, the overlap was much more evident, with 72% of the gene sets downregulated and 56% upregulated by the transgene also being regulated by the miniprotein.

To identify potential mediators of Omomyc effect, we selected 8 genes significantly regulated by both conditions (7 downregulated and 1 upregulated; Fig. 6C). These genes belong to the MYC network, regulate cell cycle, proliferation, apoptosis, migration, invasion, and metastasis, and/or are prognostic in breast cancer. We then validated their regulation by Omomyc at the mRNA level by qRT-PCR. In these conditions, the expression of five of them was clearly modulated by both the Omomyc transgene and the miniprotein (*SKP2*, *CHAF1B*, *MAD2L1*, *DEPDC1B*, and *EGR1*, although *EGR1* was not statistically significant for the transgene), one by the transgene only (*SCN5A*), and two by the miniprotein only (*YEATS4*, *TGFBI*; Fig. 6D). Notably, making use of available clinical data, we observed that all the aforementioned genes except *SCN5A*, increasing or decreasing the mRNA level in the same direction as Omomyc, could confer a significant survival advantage in patients with breast cancer (Supplementary Fig. S6D). On the same line, patients with breast cancer harboring alterations in at least 1 of the 7 downregulated genes — mainly gene amplifications (Supplementary Fig. S6E) — have a significantly shorter overall survival (Fig. 6E). Strikingly, *MYC* is the top altered gene among such patients, followed by long noncoding RNA (lncRNA) genes from the 8q24 gene desert region that have been shown to cooperate with MYC and to exert protumorigenic functions in breast and other cancers (Fig. 6F; Supplementary Table S1; refs. 68, 69).

**FIGURE 7:**
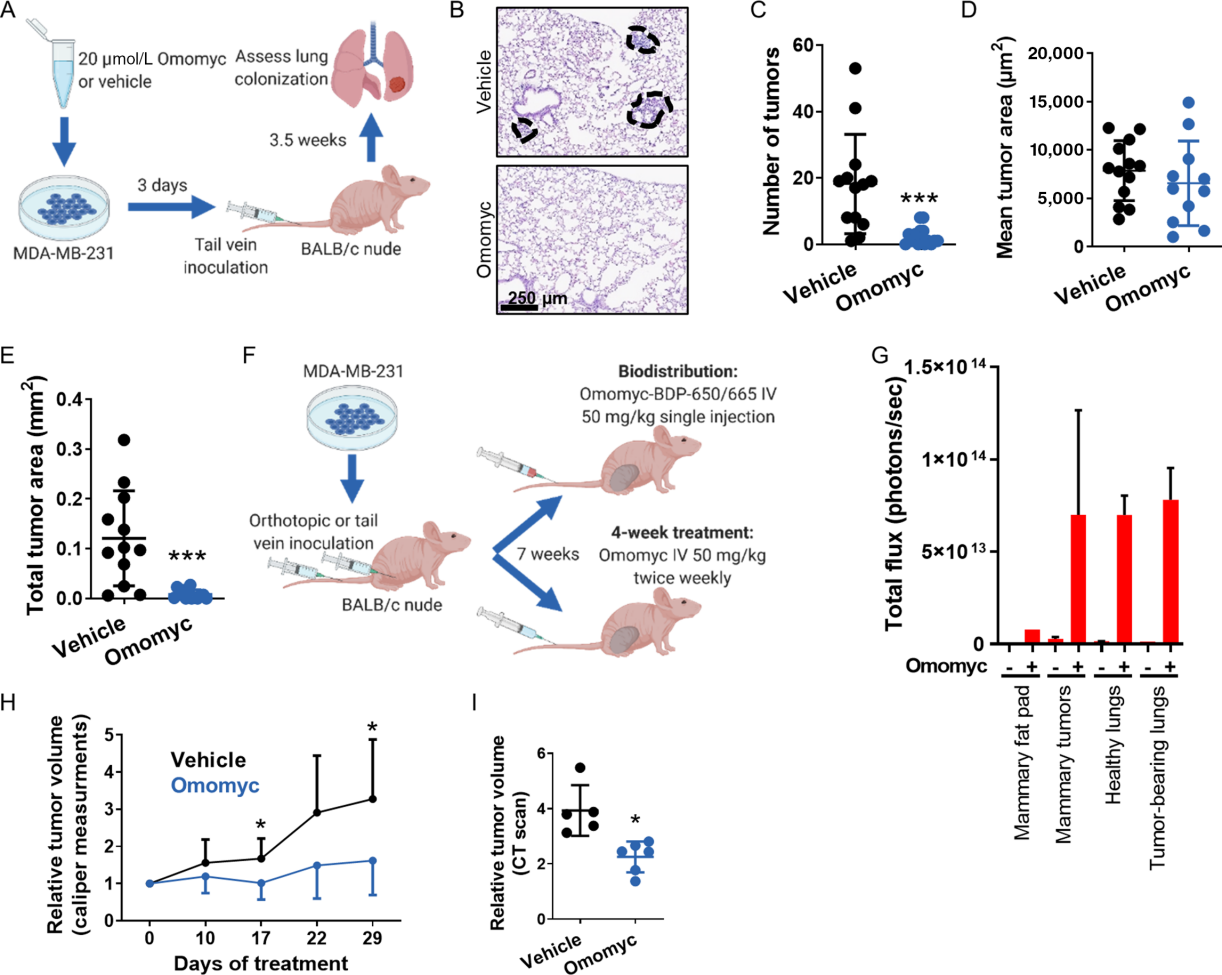
Pretreatment of MDA-MB-231 cells with the Omomyc miniprotein *in vitro* prevents subsequent lung colonization *in vivo*. Intravenous administration of Omomyc *in vivo* reaches mammary tumors and tumor-bearing lungs and reduces mammary tumor growth. **A,** Schematic representation of the lung colonization prevention experiment. Luciferase-expressing MDA-MB-231 cells were pretreated with 20 μmol/L Omomyc or vehicle for 3 days and inoculated through the lateral tail vein of BALB/c nude females. 3.5 weeks later, lung colonization was assessed. **B,** Representative images of hematoxylin and eosin (H&E)-stained lung sections from mice inoculated with vehicle- or Omomyc-treated cells as described in **A**. **C,** Quantification of the number of tumors present in the lungs of mice inoculated with vehicle- or Omomyc-treated cells from **A**. Graph shows mean ± SD; statistical significance was determined via two-tailed unpaired *t* test. **D,** Quantification of the mean tumor area of tumors present in the lungs of mice inoculated with vehicle- or Omomyc-treated cells from **A**. Graph shows mean ± SD; statistical significance was determined via two-tailed unpaired *t* test. **E,** Quantification of the total tumor area in the lungs of mice inoculated with vehicle- or Omomyc-treated cells from **A**. Graph shows mean ± SD; statistical significance was determined via two-tailed unpaired *t* test. **F,** Schematic representation of the biodistribution and efficacy experiment. Luciferase-expressing MDA-MB-231 cells were inoculated orthotopically or through the lateral tail vein of BALB/c nude females. Seven weeks later, mice received a single intravenous injection of vehicle or 50 mg/kg Omomyc-BDP-650/665. Mice bearing orthotopic tumors were also treated twice a week for 4 weeks with vehicle or 50 mg/kg Omomyc. **G,** Quantification of total fluorescence per organ in the mammary fat pad, mammary tumor, healthy lungs, or tumor-bearing lungs of mice treated with vehicle or with 50 mg/kg Omomyc-BDP-650/665, 1 hour after intravenous administration. Graph shows mean + SD. **H,** Relative tumor volume calculated from caliper measurements in mice bearing mammary tumors treated twice a week for 4 weeks with vehicle or with 50 mg/kg Omomyc. Graph shows mean + SD (vehicle) and mean − SD (Omomyc); statistical significance was determined via two-tailed unpaired *t* test. **I,** Relative tumor volume obtained by CT scans of the same mice in **H**. Graph shows mean ± SD; statistical significance was determined via two-tailed unpaired *t* test.

Encouraged by these *in vitro* results, we then assessed the antimetastatic and antitumor function of the Omomyc miniprotein *in vivo*. To do so, we initially pretreated MDA-MB-231 cells with 20 μmol/L of the Omomyc miniprotein or with vehicle for 3 days, and inoculated them through the lateral tail vein of BALB/c nude mice. 3.5 weeks later, lung sections were stained with H&E and the number and area of lung tumors calculated (schematic in Fig. 7A). Strikingly, mice inoculated with cells pretreated with Omomyc presented a dramatic reduction in the number of micrometastases (Fig. 7B and C), indicating that a short pretreatment with Omomyc was able to reduce the seeding of these cells into the lung. In addition, despite the fact that the area of each of the few micrometastases present in the lungs of mice inoculated with Omomyc-treated cells was no different from the one in control mice (to be expected, since these mice did not receive Omomyc after inoculation; Fig. 7D), the impairment in the seeding caused by Omomyc was clearly sufficient to significantly reduce the total tumor area in these mice (Fig. 7E).

Then, to determine whether the Omomyc miniprotein could be used as a drug against established metastatic TNBC, we performed both a biodistribution and an efficacy study. In the first case, we inoculated MDA-MB-231 cells either orthotopically or intravenously into BALB/c nude mice, to generate primary tumors and lung metastases, respectively. We then treated both models with a single intravenous injection of 50 mg/kg of fluorescently labeled Omomyc and assessed its localization in the target organs after 1 hour (schematic in Fig. 7F). Interestingly, at this time point, Omomyc localized in the mammary tumors (more than in the healthy mammary fat pad) and in tumor-bearing lungs (although it should be noted that micrometastases represent a very small proportion of the total lung; Fig. 7G).

In the efficacy study, instead, we treated the orthotopic model with twice weekly intravenous administrations of 50 mg/kg Omomyc for 4 weeks (schematic in Fig. 7F). Importantly, this Omomyc treatment significantly impaired tumor growth, as demonstrated by both caliper measurements (Fig. 7H) and CT scan (Fig. 7I).

**FIGURE 8:**
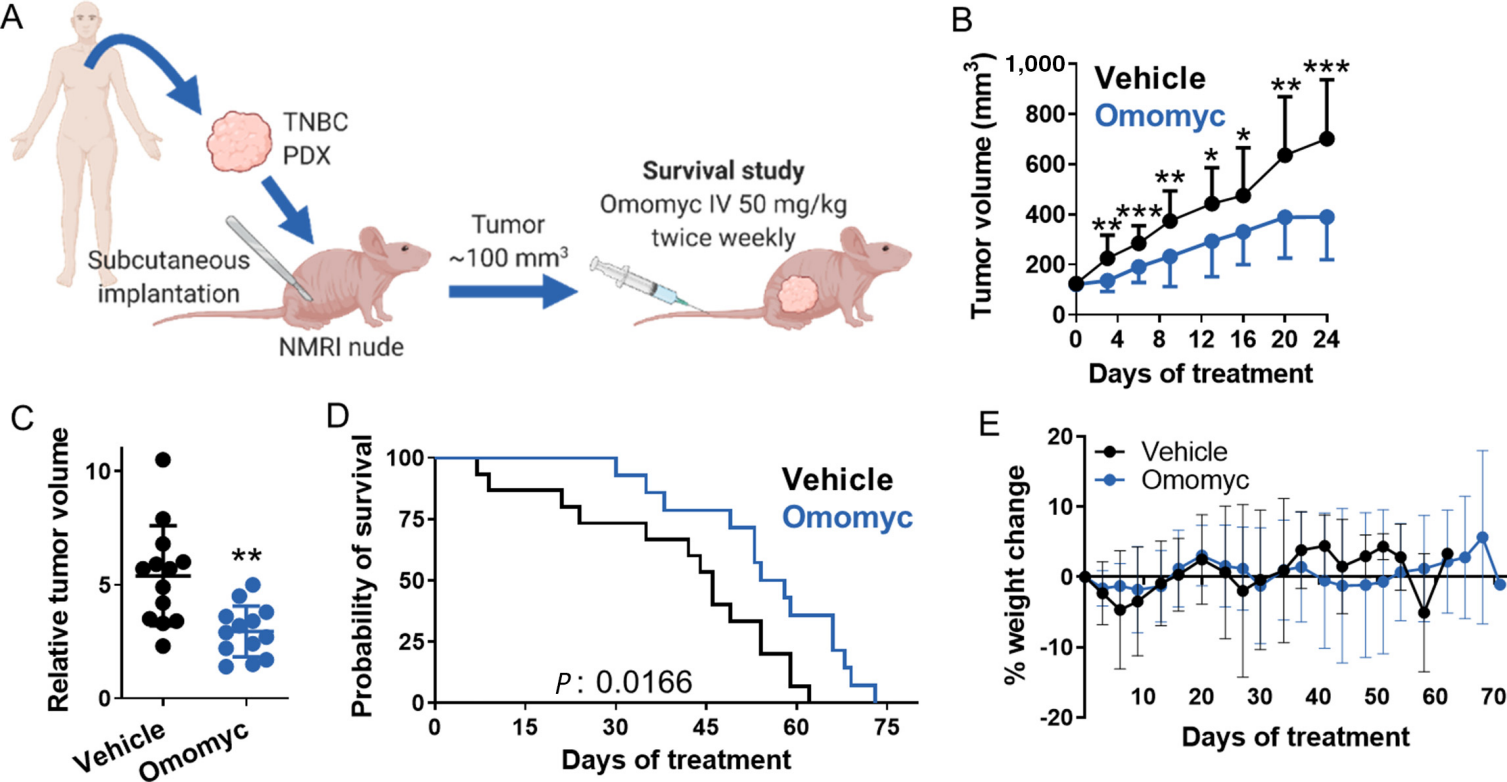
Omomyc reduces mammary tumor growth and extends survival in a TNBC PDX. **A,** Schematic representation of the PDX mouse model. Pieces from a TNBC tumor biopsy were inoculated subcutaneously into NMRI nude female mice. When tumors reached 100 mm^3^, mice were treated intravenously with vehicle or with 50 mg/kg Omomyc twice a week until they had to be euthanized (survival study). **B,** Tumor volume from vehicle- and Omomyc-treated mice during the first 24 days of treatment (when most of the animals were still under treatment). Graph shows mean + SD (vehicle) and mean − SD (Omomyc); statistical significance was determined via two-tailed unpaired *t* test. **C,** Relative tumor volume from vehicle- and Omomyc-treated mice after 20 days of treatment. Graph shows mean ± SD; statistical significance was determined via two-tailed unpaired *t* test. **D,** Kaplan–Meier survival plot from vehicle- and Omomyc-treated mice. Statistical significance was determined via log-rank (Mantel–Cox) test. **E,** Percent weight change throughout the survival study. Graph shows mean ± SD.

Finally, to further demonstrate Omomyc's potential in a more clinically relevant model, we made use of a subcutaneous TNBC patient-derived xenograft (PDX). Again, we treated tumor-bearing mice with the same dose and regimen (50 mg/kg twice-weekly) and this time, we performed a survival study (schematic in Fig. 8A). Encouragingly, treatment with Omomyc significantly reduced tumor volume (Fig. 8B and C) and conferred a survival advantage to the mice (Fig. 8D). Remarkably, treatment with intravenous Omomyc for up to 70 days did not cause any significant weight change when compared with vehicle treatment (Fig. 8E), and no indications of toxicity were observed besides the ones caused by excessive tumor burden at the experimental endpoint, consistent with the previously reported safety profile described in ref. 41.

## Discussion

The literature is populated with contradictory reports on the impact of MYC inhibition in metastasis, because there are data pointing at MYC as both inhibiting and enhancing it. Several publications associate MYC with metastasis in breast and other cancers, sometimes as a repressor (28–31), but mostly as an inducer (20–27). For instance, repression of integrin expression by MYC in breast cancer cells has been linked to suppression of cell migration and metastasis (31), while another report suggests that Skp2 cooperates with MYC to induce RhoA transcription, thereby promoting metastasis (20).

We previously demonstrated that many, perhaps all, primary tumors are addicted to MYC (18, 34–41), but we had not explored the possibility that MYC dependency is still conserved in metastases. Hence, in this study, we sought to establish the therapeutic utility of inhibiting MYC in mBC models *in vitro* and *in vivo*, with a special focus on TNBC.

In this context, we hypothesized that targeting MYC, a nonredundant node in cancer, would be effective against metastatic disease for at least two reasons:

- Metastases are genetically unstable, meaning that information from a patient's primary tumor may not accurately reflect the metastasis, and one metastasis may vary from another, hindering the benefits of targeted therapies (70). Given our data suggesting that MYC inhibition would be effective independently of the mutational profile of the tumor (33), using this approach could overcome this heterogeneity barrier.
- MYC promotes EMT and dedifferentiation, two key aspects of metastasis (13), suggesting that its inhibition could revert these features and impair the metastatic process from its very inception.

With all these data in mind and the intention of shedding some light on the matter, we first treated a panel of breast cancer cell lines and showed that Omomyc was able to reduce the clonogenic capacity of all of them, independently of their molecular subtype. Actually, the degree of response, which differed significantly among cell lines, positively correlated with the levels of transgenic Omomyc expressed in each of them, and did not correlate with MYC levels or the extent of MYC downregulation. These observations suggest that all breast cancer cell lines tested are, in principle, equally sensitive to Omomyc. This is in line with previous observations in other cancer types (38, 41) and with the idea that MYC is indispensable for the proliferation of all cancer cells. In fact, lack of correlation with MYC levels is not surprising, if we consider that cancer is more often dependent on tonic expression of MYC, rather than on its overexpression (71). It should also be noticed that MYC downregulation is not necessarily expected as a consequence of Omomyc treatment, because Omomyc inhibits MYC-dependent transcription by both sequestration of MYC away from the DNA and binding to E-boxes in the form of transcriptionally inactive dimers (Omomyc/Omomyc or Omomyc/MAX; ref. 33), both mechanisms that would not necessarily cause any downregulation of MYC levels, as opposed to other MYC-inhibitory strategies such as siRNA or degraders.

To then challenge Omomyc with the worst-case scenario in breast cancer, we used the well-characterized metastatic TNBC MDA-MB-231 cells to study the phenotypic changes induced by Omomyc expression *in vitro* and *in vivo* and its potential therapeutic impact. In this context, we showed that Omomyc induces an antiproliferative effect, blocking cell-cycle progression, consistent with MYC's well-established functions in controlling several cell cycle–related factors (72).

Then, to study Omomyc's impact on the metastatic phenotype *in vitro*, we focused on direct and indirect contributors to metastasis, such as migration and invasion of tumor cells, and promotion of angiogenesis. Regarding the latter, we observed that conditioned medium from cells expressing Omomyc heavily impaired tube formation in endothelial cells compared with control medium, consistent with MYC's role in promoting vasculogenesis and angiogenesis through regulation of angiogenic factors such as VEGF, thrombospondin-1, and angiopoietin-1 (62, 73). We also demonstrated that MYC inhibition by Omomyc caused a profound reduction in directional migration of MDA-MB-231 cells in a wound-healing assay. This effect was more pronounced in the presence of chemoattractants (Boyden chamber assay), and, above all, in the presence of ECM (Matrigel-coated Boyden chamber assay). This implies that MYC is able to promote cell motility itself, as well as directional movement in a gradient of chemoattractant factors, and that the expression of Omomyc can block these prometastatic features. The results in the presence of ECM are consistent with the previously described role of MYC in the release of ECM proteases (74–76). Furthermore, it is known that MYC inhibits the expression of E-cadherin in epithelial cells and controls the expression of many other EMT regulators, including N-cadherin and Snail (77, 78). Indeed, two independent publications confirmed this by showing the effect of Omomyc on cadherins, thus reenforcing our data. In one, Omomyc expression in lung epithelial cells harboring knock down of p53, mutant *Kras*^G12V^, and MYC was found to suppress EMT, decreasing expression of the metastasis-promoting *ZEB1* gene at the RNA level, combined with an increase in *CDH1*, the gene encoding E-cadherin (79). In the other, Omomyc downregulates GLI1, a transcription factor responsible for inducing metastatic and stem-like phenotypes in colon carcinoma cell lines (80).

Here we show that Omomyc behaved similarly *in vivo*. When challenged against primary mammary tumors, transgenic Omomyc dramatically reduced tumor expansion by reducing cell proliferation and enhancing apoptosis, in line with what had been shown in the *MMTV-Wnt1* mouse model of breast cancer (81), and with previous reports showing that indirect inhibition of MYC is extremely effective in the treatment of TNBC models (82).

However, as mentioned previously, some reports claim that MYC, while promoting primary tumor growth, impairs metastasis, suggesting that MYC inhibition reduces primary tumor growth but could enhance invasiveness (31). Therefore, an overarching goal of this project was to clarify this important aspect of MYC biology and demonstrate the utility of inhibiting MYC in the metastatic setting *in vivo*. To this end, we made use of two different metastatic models. In the lung colonization model, we assessed the capacity of Omomyc to prevent the seeding of breast cancer cells in the lung, mimicking incipient metastases found in patients with breast cancer. In this context, we showed that Omomyc significantly reduced both the establishment of micrometastases after seeding and also the growth of the fewer established lung secondary tumors. In another model, we treated established metastases after resection of the primary tumors. In this case, Omomyc unveiled its full potential by making established lesions shrink and, in some cases, even eradicating them completely.

Although Omomyc has proven efficacious against multiple mouse models of cancer, and we have shown here that it could also be employed against metastasis, its value as a viable pharmacologic approach was, until recently, still questionable. However, we recently showed that the purified Omomyc miniprotein displays cell-penetrating properties and has the innate capacity to enter NSCLC cells, reach their nuclei and interfere with MYC transcriptional activity, causing a therapeutic impact in mouse models of lung cancer (41). Here we have shown that these features are recapitulated in mouse models of breast cancer as well. More in detail, *in vitro*, we have demonstrated that the Omomyc miniprotein penetrates into breast cancer cells in a dose-dependent manner, interfering with cell proliferation, phenocopying the effects of lentivirally expressed Omomyc, and promoting cell death. We have also shown that combination with paclitaxel is very effective against TNBC cells, pointing to a potential combination therapy in the clinic.

Importantly, microarray analysis of MDA-MB-231 cells treated with either transgenic Omomyc or the Omomyc miniprotein demonstrated a high degree of overlap, especially at the gene set level. In a nutshell, treatment with either form of Omomyc switches off MYC targets and impacts on protumorigenic and prometastatic gene sets, downregulating cell-cycle progression, EMT, and genes related to breast cancer grade, and upregulating genes involved in tumor rejection, among many others. In these gene sets, we identified a panel of eight candidates among the top genes regulated by both conditions that can at least partially explain the therapeutic effect exerted by Omomyc: 7 downregulated (*SKP2*, *YEATS4*, *SCN5A*, *CHAF1B*, *TGFBI*, *MAD2L1,* and *DEPDC1B*) and 1 upregulated (*EGR1*). *SKP2* is a recognized direct MYC target and has been reported to interact with MYC to promote gene transcription and regulate RhoA, and its downregulation by shRNA inhibits lung metastasis of TNBC *in vivo* (20, 83, 84). *YEATS4* has been described as an oncogene in several cancer types, interacts directly with MYC and has been implicated in migration and invasion in breast cancer through regulation of miRNAs (85, 86). *SCN5A* codes for a subunit of a voltage-gated Na^+^-channel that is elevated in aggressive breast cancer and enhances breast cancer growth and metastatic dissemination; its silencing by siRNA in MDA-MB-231 cells reduces invasion (87, 88). *CHAF1B* is a subunit of the chromatin assembly factor-1 (CAF-1), which plays a role in DNA replication and repair; it is a proliferation marker in breast cancer and has been associated with histologic grade in breast and other cancers; its knockdown inhibits tumor growth and migration in a model of hepatocellular carcinoma (89, 90). *TGFBI* codes for a matrix protein that modulates cell–collagen interactions and has a controversial role in cancer, with studies describing both tumor-suppressing and tumor-promoting roles (91); in breast cancer in particular, in one study, *TGFBI* expression was shown to reduce cellular growth and tumorigenicity (92), in contrast with a more recent study in which *TGFBI* was related to cancer stem cells and metastasis (93). *MAD2L1* is a component of the mitotic spindle assembly checkpoint that has been linked to early metastasis in breast cancer and is associated with *BRCA1/2* pathogenic mutations (94, 95). *DEPDC1B* is upregulated in lung cancer, shows a negative correlation with patient survival and has been linked to a metastasis-related malignant phenotype (96); it has also been described as tumor- or metastasis-promoting in various other cancer types, such as glioblastoma, bladder, prostate, and pancreatic cancer (97–100). Finally, *EGR1* is a tumor suppressor that is a direct target of MYC through a noncanonical promoter; MYC is recruited to the promoter together with ARF, which is necessary for transcriptional induction of *EGR1*, and potentiates p53-independent, MYC-induced apoptosis (101, 102).

In addition to their involvement in breast cancer pathogenesis, our analysis of clinical data has also shown that these genes have a prognostic value. Furthermore, *MYC* is the most altered gene among patients harboring alterations in at least 1 of the 7 downregulated genes, followed by the lncRNAs *CASC8*, *POU5F1B*, *CCAT1*, *PVT1*, *PCAT1*, and *PRNCR1*, which not only belong the 8q24 gene locus (that contains *MYC*), commonly amplified in breast cancer, but also have been associated with promoting MYC-induced tumorigenesis (68, 69). Other altered genes in these patients are *FRS2*, *LYZ*, and *CPSF6*, all associated to breast cancer pathogenesis (103–105). For these reasons, we believe that the identification of these 8 genes is a first step towards understanding the antitumor and antimetastatic properties of Omomyc in mBC, but further validation and functional studies will be required to fully elucidate Omomyc's mechanism of action.

To translate the potential of MYC inhibition in breast cancer to a relevant clinical setting, we made use of three models of TNBC to validate the Omomyc miniprotein therapeutic impact *in vivo*. Lung colonization was heavily impaired by pretreatment of TNBC cells with a single dose of Omomyc, demonstrating that the miniprotein interferes with the seeding of circulating cells, a key aspect of the metastatic process. In addition, upon intravenous administration, Omomyc was found localized in both mammary tumors and metastasis-bearing lungs and exerted a significant therapeutic impact in cell line–derived (CDX) and PDX models, without causing any relevant side effect to the animals, demonstrating that Omomyc is a safe and effective drug against the disease. These data have contributed to the design of the MYCure clinical trial, a phase I/II study to evaluate the safety, pharmacokinetics, and efficacy of intravenous Omomyc in solid tumors (NCT04808362).

In conclusion, this work demonstrates that MYC inhibition by Omomyc is an effective therapeutic option against mBC, by impairing cell proliferation, angiogenesis, migration and invasion *in vitro*, dramatically reducing both primary tumor and metastatic growth, and, in some cases, even eradicating established metastases. Thus, we have demonstrated for the first time the applicability of Omomyc against metastasis, challenging the controversial notion that MYC inhibition could potentiate – rather than inhibit – invasion. Finally, we have validated the Omomyc miniprotein as the first directly deliverable Omomyc-based drug for the treatment of metastatic TNBC, providing a new therapeutic opportunity for patients suffering from this dreadful and incurable disease.

## Supplementary Material

Supplementary Data Supplementary Figures 1-6 and Supplementary Tables 1-2
