# Supplementary material for: MYC Inhibition Halts Metastatic Breast Cancer Progression by Blocking Growth, Invasion, and Seeding
Source: Cancer Res Commun. 2022 Feb 21;2(2):110–30. doi: 10.1158/2767-9764.CRC-21-0103 (PMC9973395; doi:10.1158/2767-9764.CRC-21-0103)
Supplement: Supplementary Data — Supplementary Figures 1-6 and Supplementary Tables 1-2 [file crc-21-0103-s01.pdf]

Massó-Vallés et al. Supplementary figures and tables

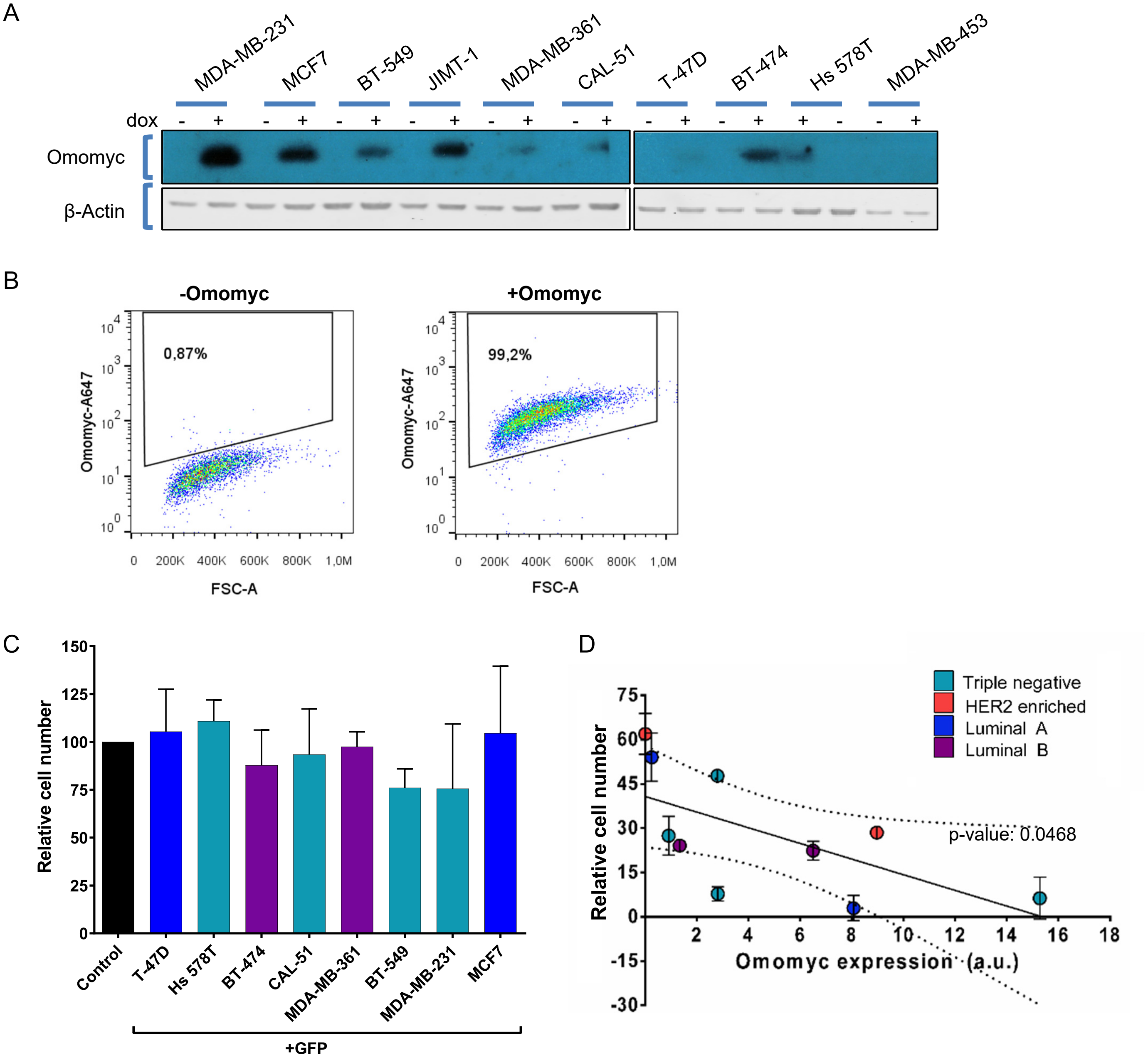

Supplementary Figure 1. Omomyc expression levels in BC cells. A. Western blot of Omomyc 3 days after addition of 0.6 µg/mL doxycycline. **B.** Representative density plots of Omomyc levels in MDA-MB-231-Omomyc cells untreated (-Omomyc) or treated with 0.6 µg/mL doxycycline for 3 days (+Omomyc) measured by flow cytometry. **C.** Quantification of relative cell number after GFP expression. Graph shows mean + SD; Statistical significance was determined via two-tailed Mann–Whitney test. **D.** Linear regression of relative cell number vs. Omomyc expression levels in each cell line. Graph shows mean + standard error of the mean (SEM); statistical significance was determined via two-tailed Pearson correlation.

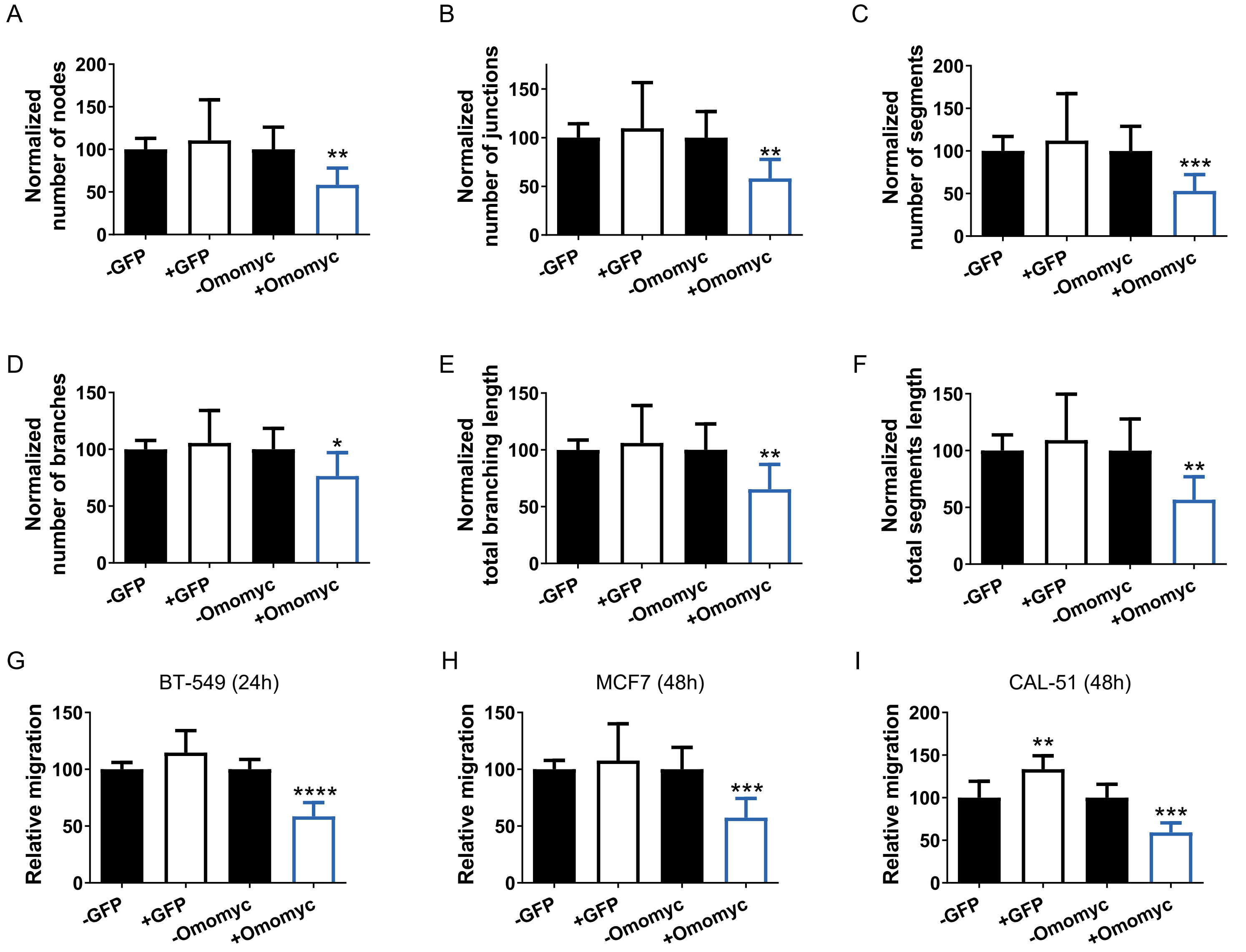

Supplementary Figure 2. Omomyc expression reduces the capacity BC cells to induce angiogenesis and to migrate. A-F. Quantification of number of nodes (**A**), number of junctions (**B**), number of segments (**C**), number of branches (**D**), total branching length (**E**) and total segments length (**F**) in HUVEC cells exposed to conditioned media from MDA-MB-231-GFP cells untreated (-GFP) or treated with 0.6 µg/mL doxycycline for 3 days (+GFP) and from MDA-MB-231-Omomyc cells untreated (-Omomyc) or treated for 3 days with 0.6 µg/mL doxycycline (+Omomyc). Graphs show mean + SD; statistical significance was determined via two-tailed unpaired T test. **G-I.** Quantification of migrated BT-549 (**G**), MCF7 (**H**) and CAL-51 (**I**) cells corrected for the total cell number in a migration Boyden chamber assay. Cells were plated on top of a Boyden chamber in 0.5% FBS and migrated for 24 or 48 hours through a porous membrane towards wells containing 10% FBS. Graphs show mean + SD; statistical significance was determined via two-tailed unpaired T test.

A

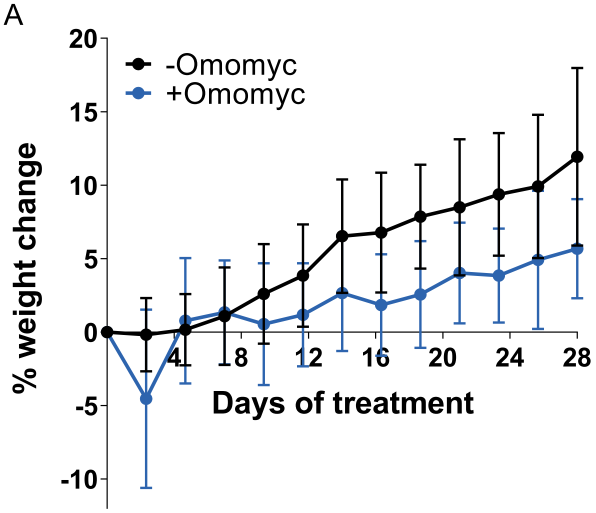

Supplementary Figure 3. Mouse weight change upon Omomyc expression. A. % of weight change in mice bearing orthotopic MDA-MB-231-Omomyc tumors treated with 5% sucrose (-Omomyc) or with 2 g/L doxycycline diluted in 5% sucrose (+Omomyc). Graph shows mean ± SD.

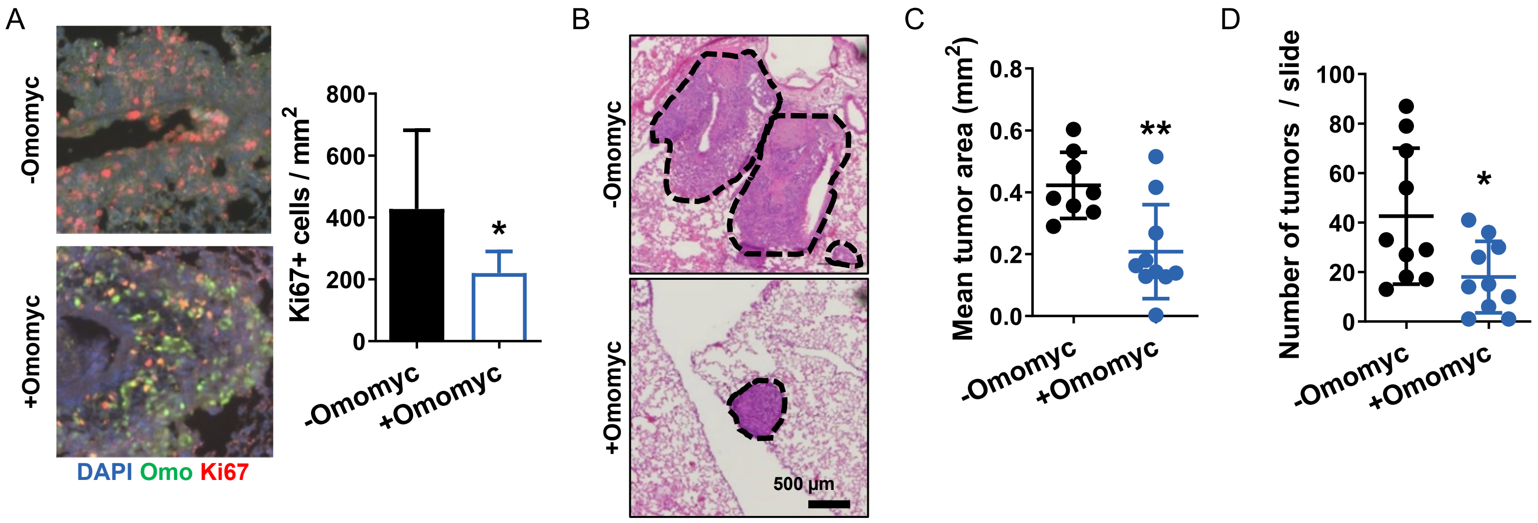

Supplementary Figure 4. Omomyc expression reduces proliferation and lung tumor burden. A. Representative images of double immunofluorescence for Omomyc and Ki67 in tumors from sucrose- and doxycycline-treated mice (left panels) with its quantification (right panel). Graph shows mean + SD; statistical significance was determined via two-tailed unpaired T test. **B.** Representative hematoxylin and eosin (H&E)-stained lung sections in a lung colonization mouse model after expression of Omomyc for 5 weeks. **C.** Quantification of the mean tumor area in the lungs of mice treated with sucrose (-Omomyc) or doxycycline (+Omomyc). Graph shows mean ± SD; statistical significance was determined via two-tailed unpaired T test. **D.** Number of tumors in the lungs of mice treated with sucrose (-Omomyc) or doxycycline (+Omomyc). Graph shows mean ± SD; statistical significance was determined via two-tailed unpaired T test.

A

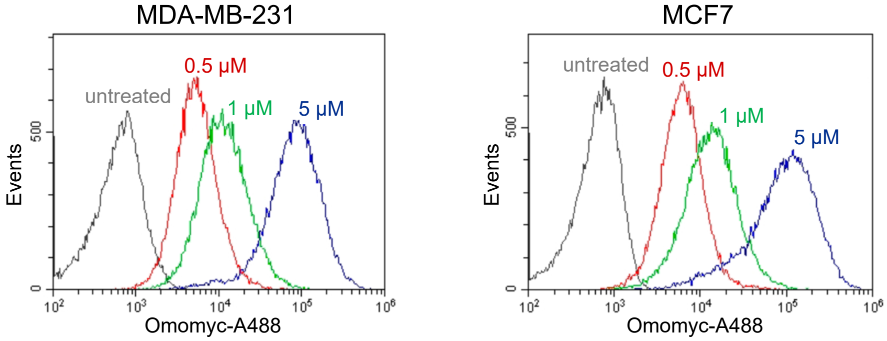

Supplementary Figure 5. The Omomyc mini-protein spontaneously penetrates into MDA-MB-2321 and MCF7 cells in a dose-dependent manner. A. Flow cytometry analysis of MDA-MB-231 and MCF7 cells untreated or treated with Omomyc-Alexa Fluor 488 (Omomyc-A488) at 0.5 µM, 1 µM and 5 µM for 15 minutes.

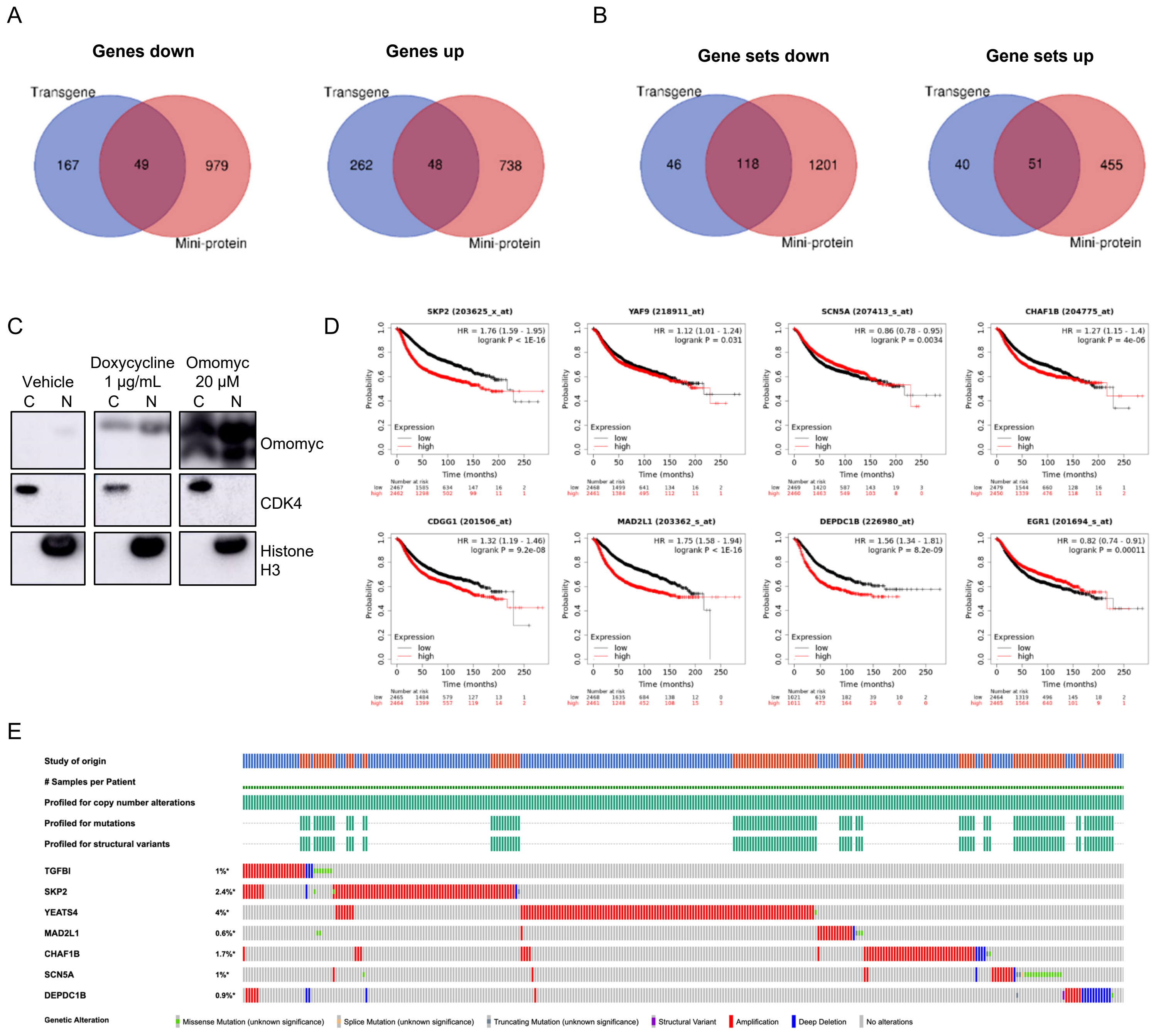

Supplementary Figure 6. Expression of the Omomyc transgene and treatment with the Omomyc mini-protein regulate common genes and gene sets in MDA-MB-231-Omomyc cells. A. Venn diagram of downregulated (left) and upregulated (right) genes after expression of the Omomyc transgene (1 µg/mL doxycycline for 4 days) or treatment with the Omomyc mini-protein (20 µM for 3 days). **B.** Venn diagram of downregulated (left) and upregulated (right) gene sets after expression of the Omomyc transgene or treatment with the Omomyc mini-protein. **C.** Western blot showing cytoplasmic (C) and nuclear (N) Omomyc after subcellular fractionation of cells expressing the Omomyc transgene or treated with the Omomyc mini-protein. CDK4 was used as a cytoplasmic marker and Histone H3 was used as a nuclear marker. **D.** Relapse-free survival (RFS) plots of 8 selected genes significantly downregulated or upregulated by expression of the Omomyc transgene and by treatment with the Omomyc mini-protein. YAF9 = YEATS4, CDGG1 = TGFBI. Source: Kaplan-Meier Plotter for breast cancer. **E.** Type and frequency of genomic alterations in *TGFBI*, *SKP2*, *YEATS4*, *MAD2L1*, *CHAF1B*, *SCN5A* and *DEPDC1B* in BC patients. Source: cBioPortal.

.

Supplementary Table 1: Top 10 genes whose expression is altered in patients with amplification on at least 1 of the 7 genes shown in Fig. 6C.

Gene	Cytoband	Amplified group	Unamplified group	Log Ratio	p-Value	q-Value	Enriched in
MYC	8q24.21	119 (37.19%)	761 (15.59%)	1.25	1.63E-19	7.02E-18	Amplified group
CASC8	8q24.21	118 (36.88%)	589 (12.07%)	1.61	1.12E-27	8.94E-26	Amplified group
POU5F1B	8q24.21	118 (36.88%)	589 (12.07%)	1.61	1.12E-27	8.94E-26	Amplified group
CCAT1	8q24.21	117 (36.56%)	570 (11.68%)	1.65	3.01E-28	2.42E-26	Amplified group
PVT1	8q24.21	116 (36.25%)	580 (11.89%)	1.61	4.53E-27	3.48E-25	Amplified group
FRS2	12q15	114 (35.63%)	3 (0.06%)	9.18	7.19E-143	5.59E-139	Amplified group
PCAT1	8q24.21	114 (35.63%)	561 (11.50%)	1.63	4.57E-27	3.49E-25	Amplified group
PRNCR1	8q24.21	114 (35.63%)	561 (11.50%)	1.63	4.57E-27	3.49E-25	Amplified group
LYZ	12q15	113 (35.31%)	0 (0.00%)	>10	7.68E-147	1.79E-142	Amplified group
CPSF6	12q15	112 (35.00%)	1 (0.02%)	>10	2.04E-143	2.38E-139	Amplified group

Supplementary Table 2: Sequences of primers used for RT-qPCR analysis.

Target gene	Forward primer	Reverse primer
SKP2	5'-TACAGAAAGAATCTCCAGAAATCAGATC-3'	5'-GGAAAAATTCCTGAAAGCAGTCA-3'
YEATS4	5'-TGAAAGACCTGTAACCCTGTATC-3'	5'-CATCATTGCTGTTGGGTCTTG-3'
SCN5A	5'-GAGCTCTGTCACGATTTGAGG-3'	5'-GAAGATGAGGCAGACGAGGA-3'
CHAF1B	5'-TGACGGTGCCTCTGACTGT-3'	5'-GGCACCGTTCTACTTCTTCAA-3'
TGFBI	5'-ATCCCAGACTCAGCCAAGAC-3'	5'-CGCTCACTTCCAGAGAGATG-3'
MAD2L1	5'-GACATTTCTGCCACTGTTGG-3'	5'-AACTGTGGTCCCGACTCTTC-3'
DEPDC1B	5'- TGGTACCCGAACACTGATGG-3'	5'- GACGGCAAAATGATGGAGCA-3'
EGR1	5'-CAGCACCTTCAACCCTCAG-3'	5'- CACAAGGTGTTGCCACTGTT-3'
GAPDH	5'- CAAGAGCACAAGAGGAAGAGAG-3'	5'- CTACATGGCAACTGTGAGGAG-3'
β-Tubulin	5'-TCTACCTCCCTCACTCAGCT-3'	5'-CCAGAGTCAGGGGTGTTCAT-3'
